# Smart Device Use and Perceived Physical and Psychosocial Outcomes among Hong Kong Adolescents

**DOI:** 10.3390/ijerph14020205

**Published:** 2017-02-18

**Authors:** Stephen Wai Hang Kwok, Paul Hong Lee, Regina Lai Tong Lee

**Affiliations:** School of Nursing, The Hong Kong Polytechnic University, Hung Hom, Kowloon, Hong Kong; 13014549g@connect.polyu.hk (S.W.H.K.); paul.h.lee@polyu.edu.hk (P.H.L.)

**Keywords:** adolescents, smart device, physical and psychosocial outcomes

## Abstract

Excessive electronic screen-based activities have been found to be associated with negative outcomes. The aim of this study was to investigate the prevalences and patterns of smart device activities and the purposes and perceived outcomes related to smart device use, and the differences in patterns of smart device activities between adolescents who did and did not perceive these outcomes. The study was a cross-sectional survey of Hong Kong primary and secondary school students. Demographic characteristics, purpose and pattern of the activities, and frequencies of the outcomes were measured. Data from 960 adolescents aged 10–19 were analyzed. Nearly 86% of the sample use smart device daily. The one-week prevalence of perceived sleep deprivation, eye discomfort, musculoskeletal discomfort, family conflict and cyberbullying victimization related to smart device use were nearly 50%, 45%, 40%, 20% and 5% respectively. More than 25% of the respondents were at risk of negative outcomes related to smart device activities for more than 1 h per day, browsing and gaming on at least 4 days per week and watching TV/movies and posting on more than 2 days per week. Their patterns of smart device activities may put a significant number of them at risk of negative outcomes.

## 1. Introduction

Use of handheld smart devices such as smartphones and tablet computers is prevalent globally. The smartphone ownership rate has been increasing rapidly in recent years [1]. In The Netherlands, the rate is around 70% in the general population and over 90% in adolescents [2]. In Switzerland, the rate in adolescents increased from around 50% to nearly 80% from 2010 to 2012 [3]. In Germany, the rate among adolescents increased from around 25% to over 70% from 2011 to 2013 [4]. In the United States, the rate in the general population increased from 35% to 56% from 2011 to 2013 [5]. More than 60% of families with young children own a smartphone, and around 40% of them own tablet computers [6]. In Asia, the smartphone ownership rate among adolescents is around 85% in South Korea, around 65% in Japan and the Philippines, over 55% in Malaysia and Hong Kong, and over 40% in China [7]. It is comparable to the smartphone ownership rate of nearly 50% among adolescents in the United States [8]. Nearly three-quarters of the U.S. teens have or have access to a smartphone [9].

A vast majority of adolescents in Hong Kong are smart device users. Over half of primary school students and over 90% of secondary school students possess smartphones [10]. Smart devices including smartphones and tablet computers are defined as handheld mobile electronic devices with cell-phone capability, having a browser that allows Internet access, a licensed operating system that provides a platform for third-party applications such as multimedia software and games, a touch screen input and output, and wireless connections that allow data transfer [11,12,13].

Media consumption via smart devices among Hong Kong adolescents may be excessive. The American Academy of Pediatrics [14] recommended restricting all children’s media time to a maximum of two hours per day, and this guideline has been adopted internationally to restrict all screen-based leisure activities using computers, electronic gaming devices and mobile phones among children [14,15,16]. In Hong Kong, over 80% of school students were regular users of smartphones, and nearly 30% of them used their smartphones for at least four hours every day [17]. Frequent and prolonged use of smart devices may increase risks of negative physical and psychosocial outcomes. These outcomes cause concern to parents, teachers and the government [10]. 

Electronic screen-based activities have been found to be related to shorter sleep duration, lower sleep quality and daytime sleepiness among adolescents. Van den Bulck [18] conducted a survey on 2546 Belgian adolescents with mean age 13.16 (SD = 0.43) and found that time spent on computer games was significantly associated with less time in bed at night. Dworak et al. [19] studied 11 German adolescents with mean age 13.45 (SD = 1.04) and found that computer gaming for 1 h before bedtime was associated with longer sleep onset latency, more stage two sleep and less slow wave sleep. Weaver et al. [20] studied 13 Australian adolescents with mean age 16.6 (SD = 1.1) and found that pre-sleep video gaming for 50 min was associated with longer sleep onset latency, reduced subjective sleepiness and change in alertness. Munezawa et al. [21] conducted a survey on 94,777 Japanese adolescents in grades 7 to 12 and found that the percentage of respondents who often or always felt excessive daytime sleepiness was 46%; the adjusted odds of sleep for less than 6 h per night, poor sleep quality and excessive daytime sleepiness related to daily mobile phone messaging after lights out were 1.15, 1.27 and 1.50 respectively. Arora et al. [22] conducted a survey on 632 UK adolescents aged 11–18 and found that mobile phone use at bedtime had a significant and negative direct effect on weekday sleep duration in path analysis. In Hong Kong, around 50% of school students always or occasionally had sleep depletion related to online activities [10]. 

Use of a handheld electronic device was found to be related to physical discomfort. About 50% of Hong Kong primary school students showed symptoms of unclear vision and felt eye strain related to the use of portable electronic devices [23]. Lui et al. [24] conducted a survey on 464 Hong Kong primary schoolchildren aged 8–13 and found that nearly 30% of the respondents reported having bodily discomfort related to electronic gaming in one month. The exposure to handheld electronic games was significantly correlated with the incidence of bodily discomfort among the schoolchildren. Two hours’ daily use of handheld devices was significantly associated with increased risk of musculoskeletal discomfort among them. Shan et al. [25] conducted a survey on 3016 Chinese adolescents aged 15–19 and found that the odds of neck or shoulder pain related to mobile phone use for over 2 h per day on average was 1.49, and the odds of low back pain related to mobile phone and tablet computer use were over 1.83.

Excessive electronic screen-based activities were found to be associated with poorer parent child relationships. Willoughby [26] conducted a survey on 1591 Canadian adolescents studying in grades 9–12 and found that time spent on computer game use was weakly associated with parental relationships. Punamäki at al. [27] conducted a survey on 478 grade 4 and 7 Finnish adolescents, and found that intensive digital game playing and Internet surfing were associated with poor relations with parents. Kwon et al. [28] conducted a survey on 1136 South Korean adolescents with mean age 14.01 (SD = 0.51) and found that Internet game addiction was significantly and positively correlated with escapism and perceived hostility in parent-child relationships. Coyne et al. [29] conducted a survey on 491 U.S. adolescents aged 12–17 and found that overall time spent on social networking was negatively correlated with the connection with parents and positively correlated with relationship aggression and delinquency. In Hong Kong, over 60% of parents had always or occasionally quarreled with their children related to use of the Internet or electronic screen products [10]. 

Online social activities were found to be related to increased risk of cyberbullying victimization. Bossler et al. [30] found that the frequency of posting sensitive information online, association with peers who harass online and maintaining social network sites significantly predicted online harassment victimization among adolescents. Dredge et al. [31] stated that self-presentation on social networking sites can increase the likelihood of eliciting negative attention from potential perpetrators. Görzig and Frumkin [32] conducted a survey on 25,142 adolescents aged 9–16 in 25 European countries and found that the odds of cyberbullying victimization on mobile phones among those who went online by using a portable handheld device and were bullied on a social networking site and on instant messaging were 1.67, 1.48 and 1.91 respectively. However, the correlation between the percentage of victimization on handheld devices among all cyberbullying victims and the percentage of handheld device use for online activities within each country was not significant. Ortega et al. [33] conducted a survey on 2227 English, 1964 Italian and 1627 Spanish adolescents with mean age 14.20 (SD = 1.77) and found that 4.1%, 9.5% and 4.2% of the respondents, respectively, reported being victimized occasionally or more frequently in cases of mobile phone cyberbullying in 2 months. Wong et al. [34] conducted a survey of 1917 Hong Kong adolescents aged 12–15 and found that 23% of the respondents reported being a victim of cyberbullying in one month. However, the percentage of victimization among Hong Kong adolescents on smart devices is unknown. 

Smart devices can be used for purposes of either academic study or leisure among adolescents. Smart device use can facilitate inquiry-based learning, such as participating in online discussions, which benefits adolescents in terms of their competence in information technology and literacy, inquiry skills, self-efficacy and critical thinking [35,36,37]. On the other hand, they may use smart devices for leisure activities such as Internet surfing, social networking, messaging, gaming and watching videos [7,38]. Excessive smart device use should be controlled, particularly if its use for leisure is more prevalent. However, there is limited evidence on the prevalence of smart device use for the purposes of academic study or leisure, or on the differences in patterns of smart device activities within a specific timeframe between adolescents who did and did not perceive the outcomes.

The aim of this study was to investigate the prevalence and patterns of smart device activities and purposes and perceived outcomes related to smart device use, and the differences in patterns of smart device activities between adolescents who did and did not perceive the outcomes. The objectives were as follows:

1. To investigate the prevalence, frequency and time spent on smart device activities, overall use and whether the purpose was academic study or leisure.
2. To examine the prevalence and frequency of perceived outcomes related to smart device use.
3. To study the differences in frequency and time spent on smart device activities between adolescents who did and those who did not perceive these outcomes.

The frequency of negative outcomes related to smart device use among adolescents might be reduced if significant relationships between smart device activities and perceived outcomes can be identified, therefore health interventions could be focused on problematic behaviors in order to reduce the risk of negative outcomes.

## 2. Methods

### 2.1. Study Design

The study design was a cross-sectional survey. It was a non-experimental design to collect data for studying the prevalences of variables and group differences in variables without manipulating the respondents. Demographics, behavioral and outcome variables were measured in the form of a questionnaire printed in traditional Chinese.

### 2.2. Sampling and Recruitment Procedure

Convenience sampling was adopted to recruit schools and their students. The inclusion criteria for schools were (1) being registered with the Hong Kong Education Bureau [39]; (2) providing education at either primary or secondary level; and (3) being a non-special school. Registered schools in Hong Kong are supposed to follow the curriculum and assessment guidelines proposed by the Hong Kong Education Bureau [40]. The recruited schools were assumed to be representative of Hong Kong primary and secondary schools in general. The inclusion criteria for adolescents were (1) being aged 10 to 19; (2) having experience in using smart devices; and (3) ability to complete the questionnaire without assistance. 

Schools were recruited via direct contact. Contact information was obtained from the school websites. School principals were invited to consent to participate in the study on behalf of the school. The consent form and information sheet were sent to principals via email. Signed consent forms were returned to the researcher before data collection. During class time at school, students were informed that those aged 10 or above were being invited to participate in the study. The consent form and information sheet were delivered to parents by their invited children. Having been informed of the study aim, giving consent to participate in this study implied that a participant had experience of smart device use in his/her lifetime. On the next day, students who returned consent forms signed by their parents were recruited. 

### 2.3. Data Collection

The data collection period was from July to October 2015. Questionnaires were prepared and delivered to schools by the researcher. In each school, the principal appointed a teacher or a vice principal to coordinate the study logistics. Class teachers were briefed on the aim, procedure and ethical issues of the study. They delivered the questionnaires to students who had returned consent forms and agreed to participate in the study. Students were asked to read the instructions on the questionnaire, put ticks in the box indicating their answers to the items, and fill in the blanks, date of completion, class name and class number on the questionnaire. Completed questionnaires were submitted to class teachers before or at the end of the school day. Class teachers placed the questionnaires in envelopes, class by class, and returned them to the researcher after school. In the study, 1690 students were invited in three primary schools and two secondary schools. 1494 of the students and their parents consented to participate in the survey. 1418 students completed and returned their questionnaires. The response rate was 83.9%.

### 2.4. Ethical Considerations and Confidentiality

The study was conducted in accordance with the Declaration of Helsinki. The study was approved by the Human Subjects Ethics Sub-committee of the Hong Kong Polytechnic University. The Reference Number was HSEARS20150629002. Permissions to conduct the study were obtained from the management committees of the recruited schools. School principals, students and parents were given consent forms and information sheets outlining the study aim, and the significance and procedure of data collection. 

The study aim, the voluntary nature of participation in the study, and participants’ right to withdraw from the study without penalty were stated in the information sheet and verbally explained to respondents before data collection. Confidentiality was ensured throughout the study. No school or student names were required on the questionnaire. Demographic information was solicited on the inside page. School staff were instructed not to read the answers on the questionnaires. Only the researchers have reviewed and analyzed the raw data. Data were not used for any other purpose than the study. Hard copies of the data were stored in a secure place, and soft copies of the data were stored in encrypted memory, both of which were only accessible to the researcher.

### 2.5. Operational Definition of Terms

1. Adolescents are young people aged between 10 and 19 [41].
2. Behavioral variables were the patterns of smart device activities, overall use, and the purpose of use for either academic study or leisure. Patterns were the frequencies and time spent on the activities, overall use and its purposes. Smart device activities were messaging [42,43,44,45,46], browsing information [45,47,48,49,50], gaming [1,38,45,51,52,53,54,55,56,57], watching TV/movies [1,45,56,58,59] and posting information [56,60,61,62] conducted on a smartphone or tablet computer.
3. Outcome variables were frequencies of the outcomes related to smart device use. The outcomes were sleep deprivation [1,10], eye discomfort [23], musculoskeletal discomfort [17,24,63,64,65], family conflict [10,54] and cyberbullying victimization [10,34] identified in the literature.

### 2.6. Demographic Measures

Age was measured using an open-ended question and rounded up to years. Gender was measured on a nominal scale of “male” and “female”. Grade was measured on an ordinal scale of “primary 4”, “primary 5”, “primary 6”, “secondary 1”, “secondary 2”, “secondary 3”, “secondary 4”, “secondary 5” and “secondary 6”, which are equal to grades 4 to 12 in the U.S. system respectively. Family monthly income was measured on an ordinal scale of “HKD 1–4000”, “HKD 4001–8000”, “HKD 8001–12,000”, “HKD 12,001–16,000”, “HKD 16,001–20,000”, “HKD 20,001–26,000”, “HKD 26,001–33,000”, “HKD 33,001–43,000”, “HKD 43,001–65,000”, and “over HKD 65,000”, which were the 10 percentiles of Hong Kong family monthly income [66]. One U.S. dollar is approximately equal to 7.75 Hong Kong dollars (Appendix A).

### 2.7. Measure Frequency

The number of days on which a smart device activity was conducted, the device was used overall or for study or leisure, or an outcome was experienced in the latest week before the survey was measured on a 5-point ordinal scale with 0 = None, 1 = 1–2 days, 2 = 3–4 days, 3 = 5–6 days and 4 = Every day. In previous studies, scales such as “Yes, No” [24] and “No or hardly ever, 1 or 2 times weekly, 3 or 4 times weekly, Most days” [67] were used to measure frequency of outcome in the latest month before the survey. Scales such as “>1 time per day, 1–6 times per week, >4 times per month” [24], “Never, Once a week or less, Twice a week, 3–4 times a week, 5–7 times a week” [1], and “None, 1–3 times/month, Once/week, Several times/week, Every day” [21] were used to measure the frequency of an activity or the device use. Recalling monthly events may involve a higher risk of bias than recalling weekly events. Options without specific and regular intervals may not allow meaningful comparisons. Therefore, recalling the frequency of events in the latest week before the survey may minimize recall bias. Relatively regular intervals in a scale may improve comparison between options as well as between respondents.

### 2.8. Measure Time Spent

Average time spent on a smart device activity or the device use per day on which the activity was conducted or the device was used in the week prior to the survey was measured on a 6-point ordinal scale with 1 = None, 2 = 1–60 min, 3 = 61–120 min, 4 = 121–180 min, 5 = 181–240 min and 6 = over 240 min. In previous studies, scales such as “>4 h, 2–4 h, 1–2 h, 0.5–1 h, <0.5 h” [24] and “None, <1 h, ≥1 and <2 h, ≥2 h” [21] were used to measure the time spent on device use. However, options in a scale should not overlap. To minimize the time gap between consecutive options, the options were set in terms of minutes. A significant number of Hong Kong children and adolescents use a smart device for more than 4 h per day [17]. The upper bound of the scale was set at more than 240 min, which is 4 h. The durations of the outcomes were not measured because it was not practical to measure and compare them objectively among respondents.

### 2.9. Data Analysis

SPSS Statistics 21.0 (IBM, Armonk, NY, USA) was used to:

1. generate descriptive statistics of the demographics, the patterns of smart device activities, the overall use and its purposes, and the frequencies of the outcomes.
2. We conducted an independent sample t test to compare the mean age between genders, and a chi-square test to compare categories of grades and percentiles of income between genders.
3. We conducted a Mann-Whitney U test to compare the mean ranks of patterns of smart device activities between adolescents who did and did not perceive the outcomes.
4. We conducted complex sample binary logistic regression to examine the likelihood of the outcomes related to smart device activities, the overall use and its purposes.

Invalid responses such as missing data or more than one choice made on an item were not input into the software. System-missing values were managed by pairwise deletion in data analysis. Respondents who failed to report age or gender that met the inclusion criteria, or who responded that the pattern of smart device use overall was less than the pattern of any smart device activity or purpose of device use, were excluded from analysis. A final sample of 960 respondents proceeded to data analysis.

The Mann-Whitney U test was also adopted to study the relationships involving ordinal data. The significance level was set at 0.01 to reduce the risk of type I error, therefore test results with *p* < 0.01 were considered significant.

SPSS Complex Sample was used to address the clustering effect of schools. Primary schools and secondary schools were analyzed separately. There was no probability proportional to size (PPS) sampling because convenience sampling was adopted to recruit clusters and respondents. Therefore equal probability sampling was assumed. 

There were only three out of 528 local primary schools [68] and two out of 476 local secondary schools [69] being sampled which were less than 5% of the school populations. Still, there was finite population correction (FPC) in estimation for sampling without replacement (WOR). Population sizes of strata including gender in each grade varied. Sample weights of strata were calculated according to the census data [70].

There were no obvious normal distributions of responses in histograms and Q-Q plots. Regarding items measuring the purposes, activities or outcomes, the percentages of univariate outliers ranged between 2.5% and 22.1% in 12 items and between 5% and 22.2% in seven items respectively in the included primary school students and secondary school students.

The smart device activities, the overall use and its purposes were measured at ordinal level. In the test of parallel lines, there were either zero −2 log likelihood in the general model or no convergence of logit models. The proportional odds assumption was not fulfilled.

Therefore categories denoting number of days other than none of the days were collapsed into a single category in a dichotomous scale measuring an outcome. Binary logistic regression was used to examine the likelihood of the outcomes related to smart device use. Adjusted chi-squared tests of model effects were adopted to analyze clustered data [71]. Sequential Bonferroni method was used to adjust for *p* value because it is considered more powerful than traditional Bonferroni method [72]. The significance level was 0.05.

Demographics having associations with outcome variables by Spearman’s rho ≥0.2 were identified as covariates. The criteria of multicollinearity among purposes, activities and overall use of smart device were variance inflation factor >10 and bivariate Spearman’s rho ≥0.7 [73].

Purposes, activities and overall use of smart device having significant associations with outcome variables in Spearman’s correlation were included in a regression model when they met the criterion of variance inflation factor ≤10 with covariates controlled; had stronger associations with an outcome among those showing multicollinearity with Spearman’s rho ≥0.7; and contributed to a model with the least number of variables showing quasi-complete separations. 

Cases were identified as outliers and excluded from analysis when the absolute values of standardized residuals were larger than 2.5 with covariates controlled in binary logistic regression [74]. Linearities between independent variables and log odds were checked with Box-Tidewell method [75]. Variables with significant interactions with their natural logs in the models were excluded from analysis. 

Among primary school students, frequencies and time spent on activities, frequencies of leisure and overall use of smart device, time spent on leisure and overall use, and time spent on leisure use and gaming showed multicollinearity with Spearman’s rho ≥0.7. Only grade was identified as covariate of cyberbullying victimization, however, none of the purposes, activities or overall use of smart device showed significant association with the victimization. Two and three outlying cases in the regression models of musculoskeletal discomfort and family conflict were excluded from analysis respectively. Time spent on study use and frequency of gaming were excluded from the models of eye discomfort and family conflict respectively because of non-linearity.

Among secondary school students, frequencies and time spent on watching TV/movie, posting and gaming, and time spent on leisure and overall use of smart device showed multicollinearity with Spearman’s rho ≥0.7. Age and grade were identified as covariates of sleep deprivation and were controlled in the regression model. Seven and thirty-three outlying cases in the models of family conflict and cyberbullying victimization were excluded from analysis respectively. Frequency of browsing was excluded from the model of cyberbullying victimization because of quasi-complete separation.

## 3. Results

### 3.1. Demographic Characteristics

There were 960 respondents in the final sample (Table 1). Ages ranged from 10 to 19, the mean was 13.80 and the standard deviation (SD) was 1.93. In the sample, 52.6% were male and 47.4% were female. According to the Hong Kong Census and Statistics Department [66], the estimated Hong Kong male to female ratio was 1.06 for both age groups of 10–14 and 15–19 in 2015. In the sample of this study, the male to female ratio was 1.11, which was close to the estimation. Respectively, 4.7%, 8%, 16%, 14.4%, 17.2%, 20.4%, 15.2% and 4.1% of respondents were studying in grades 5 to 12. Ages and grades roughly followed normal distribution in histograms. There were 650 respondents who reported family monthly incomes and 5.4%, 4.2%, 9.1%, 10.3%, 5.8%, 9.4%, 11.1%, 10.8%, 16.2% and 17.8% of them belonged to the 1st to 10th percentiles respectively, while over 50% of them were in the highest four percentiles. There were no significant differences between genders in age, grade or family monthly income.

### 3.2. Patterns of Smart Device Activities, Overall Use and Its Purposes

Patterns of smart device activities, overall use and its purposes were measured in terms of days in a week and minutes per day on average. Among the respondents, 0.6%, 2.3%, 4.9%, 6.3% and 85.9% of them used a smart device overall for none of the days to every day respectively, and 0.6%, 11.3%, 23.6%, 20.2%, 14.5% and 29.8% of them spent no time to over 240 min per day respectively on smart device use overall (Table 2). Nearly 65% of the sample used a smart device for more than 2 h per day overall. Over 80% of the sample used a smart device for leisure on at least 5 days in a week, and nearly 50% spent more than 2 h per day using a smart device for leisure. Over 35% the respondents used a smart device for study on at least 5 days in a week, and nearly 15% spent more than 2 h per day using a smart device for study. Over 65% of the respondents used messaging and nearly 50% browsed information and played games on at least 5 days in a week respectively. Nearly 35% of the sample watched TV/movies and 25% posted information on at least 5 days in a week. Around 25% of the respondents used messaging, browsed information, played games and watched TV/movies for more than 2 h per day respectively. Nearly 15% of the respondents posted information for over 2 h per day.

There were significantly higher percentages of those who use smart device for more than 2 days per week overall, for study, leisure, posting and particularly messaging and browsing in secondary school students than the percentages in primary school students (Table 3).

There were significantly higher percentages of those who spent more than 2 h per day on smart device use overall, for study, leisure, posting and particularly messaging and browsing in secondary school students than the percentages in primary school students (Table 4). 

### 3.3. One-Week Prevalence of Perceived Outcomes Related to Smart Device Use

The five outcomes measured in relation to smart device use were sleep deprivation, eye discomfort, musculoskeletal discomfort, family conflict and cyberbullying victimization. The number of days on which an outcome was experienced in the week prior to the survey was measured. Nearly 49%, 46%, 37%, 21% and 5% of the respondents experienced sleep deprivation, eye discomfort, musculoskeletal discomfort, family conflict and cyberbullying victimization respectively on at least one day each week (Table 5). Nearly 27%, 14%, 14%, 7% and 3% of the respondents experienced sleep deprivation, eye discomfort, musculoskeletal discomfort, family conflict and cyberbullying victimization respectively on at least 3 days in a week. More adolescent smart device users perceived physical outcomes than psychosocial outcomes such as family conflict and cyberbullying victimization.

### 3.4. Differences in Patterns of Smart Device Activities Between Adolescents Who Did and Did Not Perceive The Outcomes

The Mann-Whitney U test was used to analyze the differences in patterns of smart device activities between adolescents who did and did not perceive the outcomes (Table 6). The patterns were measured by number of days in a week and minutes per day on average. Among the significant results, the mean ranks of the frequencies or time spent on smart device activities among adolescents who perceived the outcomes were higher than for those who did not. 

Adolescents who perceived sleep deprivation, family conflict or cyberbullying victimization spent significantly more time per day on messaging (*z* = −2.71 to −4.40), browsing information (*z* = −3.73 to −5.65), gaming (*z* = −3.37 to −3.82) and watching TV/movies (*z* = −3.30 to −5.81), with larger medians than those who did not perceive these outcomes. Those who perceived cyberbullying victimization also spent significantly more time per day on posting information (*z* = −4.04).

Adolescents who perceived eye and musculoskeletal discomfort spent significantly more time per day on browsing information (*z* = −3.52 to −5.96) and watching TV/movies (*z* = −2.77 to −5.92) with larger medians than those who did not perceive these outcomes. Those who perceived musculoskeletal discomfort also spent significantly more time per day on messaging (*z* = −4.36). 

Adolescents who perceived musculoskeletal discomfort and sleep deprivation browsed information (*z* = −4.00 to −4.13) and watched TV/movies (*z* = −3.69 to −4.17) on significantly more days in a week and with larger medians than those who did not perceive these outcomes. 

Adolescents who perceived family conflict and cyberbullying victimization spent time on gaming (*z* = −2.81 to −3.85) on significantly more days in a week and with larger medians than those who did not perceive these outcomes. Adolescents who perceived family conflict spent time gaming (*z* = −3.85) and watching TV/movies (*z* = −3.27) on significantly more days in a week and with larger medians than those who did not perceive the outcome. Adolescents who perceived cyberbullying victimization spent time on browsing (*z* = −3.09) and posting information (*z* = −3.68) significantly more days in a week and with larger medians than those who did not perceive the outcome. 

### 3.5. Complex Sample Binary Logistic Regression Estimating Likelihood of Outcomes of Smart Device Use

Estimates of binary logistic regression in a complex sample of primary school students were shown in Table 7. More time spent on smart device use overall was significantly associated with higher odds of family conflict (*OR* = 3.40, 95% CI (1.07, 10.82), *p* = 0.02). Higher frequency of study use was significantly related to lower odds of eye discomfort (*OR* = 0.65, 95% CI (0.48, 0.88), *p* = 0.001) and family conflict (*OR* = 0.37, 95% CI (0.12, 1.12), *p* = 0.04) respectively. More time spent on gaming was significantly related to lower odds of musculoskeletal discomfort (*OR* = 0.48, 95% CI (0.32, 0.72), *p* < 0.001).

Estimates of binary logistic regression in a complex sample of secondary school students were shown in Table 8. More time spent on smart device use overall was significantly associated with higher odds of musculoskeletal discomfort (*OR* = 1.15, 95% CI (0.99, 1.34), *p* = 0.03) and sleep deprivation (*OR* = 1.14, 95% CI (1.00, 1.29), *p* = 0.02) respectively. Higher frequency of leisure use was significantly associated with higher odds of family conflict (*OR* = 1.31, 95% CI (1.06, 1.61), *p* = 0.004) and sleep deprivation (*OR* = 1.29, 95% CI (1.01, 1.66), *p* = 0.02) respectively.

More time spent on watching TV/movie was significantly associated with higher odds of musculoskeletal discomfort (*OR* = 1.29, 95% CI (1.17, 1.41), *p* < 0.001), eye discomfort (*OR* = 1.09, 95% CI (1.01, 1.18), *p* = 0.01), sleep deprivation (*OR* = 1.28, 95% CI (1.13, 1.46), *p* < 0.001) and family conflict (*OR* = 1.24, 95% CI (1.08, 1.43), *p* < 0.001) respectively. 

More time spent on browsing was significantly associated with higher odds of musculoskeletal discomfort (*OR* = 1.36, 95% CI (1.12, 1.64), *p* < 0.001), eye discomfort (*OR* = 1.20, 95% CI (1.04, 1.38), *p* = 0.004) and family conflict (*OR* = 1.22, 95% CI (1.00, 1.50), *p* = 0.03) respectively. 

More time spent on posting was significantly associated with higher odds of cyberbullying victimization (*OR* = 2.43, 95% CI (0.91, 6.48), *p* = 0.04). On the other hand, more time spent on messaging was significantly related to lower odds of eye discomfort (*OR* = 0.88, 95% CI (0.80, 0.97), *p* = 0.002) and family conflict (*OR* = 0.85, 95% CI (0.75, 0.97), *p* = 0.01) respectively. More time spent on gaming was significantly related to lower odds of musculoskeletal discomfort (*OR* = 0.83, 95% CI (0.73, 0.94), *p* = 0.001).

Age was controlled in the model of sleep deprivation in the complex sample of secondary school students. Older age was significantly associated with higher odds of sleep deprivation (*OR* = 1.23, 95% CI (1.11, 1.35), *p* < 0.001).

## 4. Discussion

### 4.1. Prevalence of Smart Device Use

A majority of adolescents are daily smart device users. In this study, over 85% of the respondents used a smart device every day, and nearly 65% of them used a smart device for more than 2 h per day overall. The duration was longer than the limit recommended by the American Academy of Pediatrics [14], which has been adopted internationally [14,15,16]. Haug et al. [76] conducted a survey on 1519 Swiss youth aged from 15 to 21 years old and found that 97.6% of them owned a smartphone and 51% reported using it for more than 2 h per day. The high prevalence of smart device use among adolescents is worthy of concern among parents, teachers, school nurses, social workers and governments.

Excessive smart device use for leisure is more prevalent than its use for study among adolescents. In this study, over 35% of the respondents used a smart device for study on at least 5 days in a week, and nearly 15% spent more than 2 h per day using a smart device for study. However, over 80% of them used smart devices for leisure on at least 5 days in a week, and nearly 50% spent more than 2 h per day on a smart device for leisure. Smart device use for leisure may be a more significant risk factor of negative outcomes than smart device use for study. Public awareness of the risks of smart device use and behavioral control of their use for leisure may be required. 

Most adolescents conduct some type of smart device activity in a week and probably engage in multitasking according to the results of this study. The overall percentage of smart device use on none of the days in the previous week was 0.6%; however, the percentages of conducting smart device activities on none of the days were greater than 0.6%. This indicates that most adolescents conduct some type of smart device activity at least once in a week. On the other hand, the percentage of daily smart device use overall was 85.9%. However, the sum of the percentages of smart device activities conducted daily was larger than 85.9%, which may indicate multitasking in smart device use. Mak et al. [77] conducted a survey on 762 Hong Kong adolescents aged 12–20 and found that the sum of self-reported daily time spent on different screen-based devices was over 24 h in some respondents. The sum would not equal general daily time spent on screen-based devices, given that multitasking is occurring and periods of using different devices overlapped. For example, adolescents may use a device to download or play music, while simultaneously looking at the screen of another device.

### 4.2. Smart Device Activities and Negative Outcomes

#### 4.2.1. Sleep Deprivation

Sleep deprivation measured in this study is the subjective feeling of the respondent relating to smart device use. It is a significant problem among adolescent smart device users. In this study, the one-week prevalence of sleep deprivation related to smart device use was nearly 50% among the respondents. Nathan and Zeitzer [78] conducted a survey on 202 U.S. adolescents aged 14–19 and found that the prevalence of daytime sleepiness was 25% measured using the Epworth Sleepiness Scale (ESS) [79], which consists of Likert scales measuring the chance of dozing in eight circumstances. Mak et al. [77] conducted a survey of 762 Hong Kong adolescents aged 12–20 and found that the prevalence of daytime sleepiness measured by the ESS was 17.6%. The inconsistency of the prevalence rates was probably related to the construct being measured from different perspectives and contexts.

Gaming for more than one hour per day for a week may increase risk of sleep deprivation according to the results of this study (Table 6). Wolfe et al. [80] conducted a study on 21 Australian adolescents aged 15–20 and found that time spent on console games before sleep was negatively and significantly correlated with sleep duration. However, Lemola and Perkinson-Gloor [1] found that the correlation between sleep duration and frequency of video gaming was less than with other electronic media use in bed. These results were consistent with the findings in this study, that time spent on gaming among those who perceived sleep deprivation was significantly higher than among those who did not, and there was no significant difference between the two groups in the frequency of gaming.

Browsing information on more than 4 days in a week and watching TV/movies on more than 2 days in a week may increase risk of sleep deprivation. In this study, the median frequency of browsing information and watching TV/movies was 5–6 days and 3–4 days in a week respectively, and the median time spent on messaging, browsing information and watching TV/movies was 61–120 min per day among those who perceived sleep deprivation (Table 6). Regression analysis showed that higher frequency of leisure use, time spent on watching TV/movie and overall use of smart device were significantly associated with higher odds of perceived sleep deprivation with age controlled among secondary school students (Table 8). The results were consistent with the findings of a survey conducted by Lemola and Perkinson-Gloor [1] on 362 Swiss adolescents aged 12–20. They found that among adolescents who owned smartphones, the average time spent on the Internet and Facebook was 2 h and nearly 1 h respectively, and, significantly, they went online for social activities, used messaging applications and watched TV/videos in bed before sleep more frequently in a week, as well as spending more time on the Internet and Facebook on weekdays. Sleep duration on weekdays was significantly and negatively correlated with the frequencies of media use, texting, social networking and watching TV/videos in bed before sleep. 

There were some previous studies on the relationship between mobile phone use and sleep duration, sleep quality and daytime sleepiness. Arora and Broglia [81] conducted a survey of 738 UK adolescents aged 11–13 and found that frequency of mobile phone use before going to bed was significantly associated with shorter weekday sleep duration. Mak and Wu [77] found that self-reported daily mobile phone viewing duration was negatively correlated with sleep quality and sleep duration, positively correlated with daytime sleepiness, and significantly predicted poorer sleep quality and higher daytime sleepiness in a regression model with some sociodemographics being controlled. Mobile phone use before sleep may decrease sleep duration and quality and increase daytime sleepiness. However, how long adolescents should refrain from electronic screen-based activities before sleep in order to promote positive sleep outcomes is not known. Further study is required on the definition of the end time of electronic screen-based activities, bedtime, lights-out time and sleep onset; the measurement of daily periods of electronic screen-based activities and sleep; and the relationship among the periods, the time points, the timespan between the time points and sleep outcomes.

#### 4.2.2. Eye Discomfort

Eye discomfort is a significant problem among smart device users, including adolescents. In this study, the one-week prevalence of eye discomfort related to smart device use was over 45% among the respondents. There is limited study on eye discomfort related to smart device use among adolescents. Further study is required on the relationship between types and patterns of eye discomfort and smart device activities. 

Browsing information and watching TV/movies for more than one hour per day may increase the risk of eye discomfort. In this study, the medians of time spent on browsing information and watching TV/movies were 61–120 min per day among those who perceived eye discomfort (Table 6). More time spent on browsing and watching TV/movie were significantly associated with higher odds of eye discomfort in secondary school students (Table 8). Reduced blink rates during screen viewing increase cornea exposure to the air, which contributes to a poor tear film quality and temporary stresses the cornea, resulting in dry eyes [82]. On the other hand, the focusing system of the eyes locking onto a near distance on the screen may lead to accommodation spasms [83] and small temporary myopic shifts [82]. Close viewing distances from the visual display terminal place increased demands upon the ocular functions and exacerbate visual symptoms. Screen viewing for more than 4 h per day was found to be significantly associated with asthenopia [84]. 

This study found no significant differences in time spent on messaging and gaming between those who perceived eye discomfort and those who did not (Table 6). An interesting finding was that more time spent on gaming and messaging did not related to higher odds of eye discomfort (Table 8). Messaging and gaming may be more intermittent than browsing information and watching TV/movies. Further study is required on types of smart device activities and methods of measuring them.

#### 4.2.3. Musculoskeletal Discomfort

A significant number of adolescents are probably suffering from musculoskeletal discomfort related to smart device use. Among the respondents in this study, the one-week prevalence of musculoskeletal discomfort related to smart device use was over 35%. Some studies [85,86] reported prevalence of musculoskeletal discomfort in body parts separately, without reporting the overall prevalence of musculoskeletal discomfort. Therefore, the size of population suffering from musculoskeletal discomfort may not be estimated, assuming that subgroups of population may have different combinations of discomfort.

Browsing information on more than 4 days in a week and watching TV/movies for more than 2 days in a week may increase risk of musculoskeletal discomfort. In this study, the median frequency of browsing information and watching TV/movies was 5–6 days and 3–4 days in a week respectively, and the median time spent on messaging, browsing information and watching TV/movies was 61–120 min per day among those who perceived musculoskeletal discomfort (Table 6). More time spent on browsing, watching TV/movie and overall use of smart device were significantly associated with higher odds of musculoskeletal discomfort in secondary school students (Table 8).

Findings from previous studies supported that time spent on mobile electronic device use is related to musculoskeletal discomfort. Guan and Fan [87] used photogrammetry to study 186 Chinese adults and found that head tilt angle increased and neck tilt angle decreased significantly during mobile phone viewing. Straker and Burgess-Limerick [88] stated that an increase in cervical muscle activity is evident with lower display placement when using electronic products. This can potentially cause sustained muscular contraction with continual recruitment of particular motor units for long periods of time, such that localized fatigue and injury occur. 

Lui and Szeto [89] used surface electromyography to study 14 adolescents with mean age 12.29 (SD = 1.38) and found higher neck muscle activity among those using small handheld game devices. Straker and Coleman [90] stated that younger children have a proportionally greater head mass, therefore they have a higher risk of neck discomfort because of the higher gravitational load of the head away from the corresponding center of rotation and the slightly higher neck muscle activity level needed to control the head position. However, there was no significant difference in frequency and time spent on gaming between those who perceived musculoskeletal discomfort and those who did not in all included respondents (Table 6). An interesting finding was that higher time spent on gaming was significantly related to lower odds of musculoskeletal discomfort in both primary and secondary school students (Table 7 and Table 8). Behavioral pattern of gaming may be different from some other activities such as watching TV/movie and browsing. Further study is required to explore other factors that predispose adolescents to musculoskeletal discomfort related to smart device use.

#### 4.2.4. Family Conflict

Family conflict was identified as one of the problems related to smart device use among adolescents. Among the respondents in this study, the one-week prevalence of family conflict related to smart device use was nearly 21%. Gaming on more than 4 days and watching TV/movies on more than 2 days in a week may be associated with higher risk of family conflict. In this study, the medians of frequency of gaming and watching TV/movies were 5–6 days and 3–4 days in a week respectively, and the medians of time spent on gaming and watching TV/movies were 61–120 min per day among those who perceived family conflict (Table 6). More time spent on smart device use overall in primary school students, and higher frequency of leisure use and more time spent on browsing and watching TV/movie in secondary school students, were significantly associated with higher odds of family conflict (Table 7 and Table 8). The results were consistent with previous findings [27,28]. Chory and Banfield [91] found that higher levels of video game and TV dependence predicted lower use of all strategies for relationship maintenance in reality among youth. Therefore, excessive gaming and watching TV/movies may hinder positive development of interpersonal relationships and increase risk of conflict. 

Messaging and browsing information for more than one hour per day for a week may be associated with higher risk of family conflict according to the results in this study (Table 6). However, more time spent on messaging has been found significantly related to lower odds of family conflict in secondary school students (Table 8).

Although time spent online may displace in-person time with parents [92], mobile online technologies provide opportunities for connections between parents and children when separated [93], and shared use of the technologies for learning and playing may foster stronger ties through more frequent contact [94]. However, Lee [95] found that online communication did not predict child-reported quality of parent-child relationships.

Family conflicts may be associated with poorer parent-child relationships and communication online. Parents who reported frequently calling their child when they were upset or for the purposes of monitoring reported less knowledge of their child’s activities and poorer parent-child interactions than those who relied on adolescent-initiated contact [96,97]. Frequency of parental calls was also found to be negatively related to adolescent-reported truthfulness [96]. 

Secondary school students probably spent more time on smart device use for social purpose than primary school students did (Table 3 and Table 4). Yet, adolescents were found primarily to use instant messaging for much of their online interaction and for most of their communication with friends from their offline lives [98]. Further study is required on the role of smart device activities in promoting the parent-child relationship in adolescent development.

Further study is required on gender difference in family relationship relating to smart device use. A higher number of male in the entire sample may explain the significantly higher mean rank of time spent on gaming and messaging among those who perceived family conflict than those who did not (Table 6). Among all included respondents, the percentage of students who report family conflict in male was higher than the percentage in female. Time spent on gaming among male was higher than in female (χ^2^ (5, *n* = 953) = 147.91, *p* < 0.001). Among all included respondents in primary schools, the percentage of those who report family conflict in male was higher than the percentage in female. The percentage of those who spent more than one hour on messaging in male was significantly higher than the percentage in female (χ^2^ (5, *n* = 121) = 20.31, *p* = 0.001), while the difference in primary school students was larger than in secondary school students.

#### 4.2.5. Cyberbullying Victimization

Cyberbullying victimization was identified as one of the problems related to smart device use among adolescents. In this study, the one-week prevalence of cyberbullying victimization related to smart device use was nearly 5% among the respondents. The weekly prevalence in Hong Kong [34] was lower than that in China. Lam and Cheng [99] conducted a survey on 1278 Chinese adolescents aged 13–18 and found that 14.4% of the respondents reported being victimized in cyberbullying behavior in one week, as measured by a 6-item instrument. The prevalence for one month or more in Hong Kong [34] was similar to that in the U.S. but lower than that in China. Hinduja and Patchin [100] conducted a survey of 1963 U.S. adolescents aged 10–16 and found that 29.4% of the respondents reported being victimized by one or more types of cyberbullying more than once in one month, as measured by a 9-item instrument. Zhou and Tang [101] conducted a survey of 1438 Chinese adolescents with mean age 15.91 (SD = 1.02), in which 56.9% of the respondents reported being victimized in cyberbullying in the previous 5 months, as measured by an 18-item instrument. The prevalence for one month or more in some European countries [33] were lower than in Hong Kong, China and the US. Låftman and Modin [102] conducted a survey of 22,544 Swedish adolescents aged 15–18 and found that 4.8% of the respondents reported being victimized in cyberbullying during the school year in which they responded to the survey.

The inconsistency of the prevalences was probably related to differences in culture, the instrument items and the periods they measured. The prevalence of cyberbullying victimization in Europe was generally lower than in Hong Kong, China and the U.S. In a majority of instruments, the items measure frequencies of circumstances that are defined as cyberbullying by the author but not by the respondent; however, responses with regard to the frequencies were used to estimate the prevalence of cyberbullying. The prevalence may be positively correlated with the number of items in the instrument and also the period being measured. Further study is required on the definition of cyberbullying, the ideal period to measure, and the related cultural factors. 

Gaming and browsing information every day and posting information on more than 2 days in a week may be associated with higher risk of cyberbullying victimization. In this study, the median frequency of gaming and browsing information was every day, that of posting information was 3–4 days per week, and the median time spent on smart device activities was 61–120 min per day among those who perceived cyberbullying victimization (Table 6). 

Adolescents’ social development and behaviors may be associated with higher risk of cyberbullying victimization. Rice and Petering [103] conducted a survey on 1285 U.S. adolescents with mean age 12.3 (SD = 0.8), and found that the odds of cyberbullying victimization was 2 among those who texted 50 times or more per day. Time spent on gaming and messaging was found to be significantly higher among those who perceived cyberbullying victimization than among those who did not in this study (Table 6). However, only more time spent on posting was significantly associated with higher odds of cyberbullying victimization in secondary school students (Table 8). As social skills develop during psychosocial development, older adolescents may be more vulnerable to non-physical bullying [104]. On the other hand, friendship could be developed via electronic game [105]. Adolescents have also been found using messaging primarily to communicate with friends [98].

Frequency and time spent on posting and browsing information may be associated with higher risk of cyberbullying victimization. Whittaker and Kowalski [106] found that online aggressive comments directed toward peers were perceived to be significantly more negative than comments targeted toward strangers. Peluchette and Karl [107] found that posting indiscreet or negative content online, having Facebook friends who post such content, and the number of Facebook friends were strong predictors of cyberbullying victimization. The frequency of browsing information was found to be significantly higher among those who perceived cyberbullying victimization than among those who did not in this study. Sampasa-Kanyinga and Hamilton [108] conducted a survey on 5329 Canadian adolescents aged 11–20 and found that the odds of cyberbullying victimization increase along with time spent on social networking. Therefore, posting and browsing information for social activities may increase risk of cyberbullying victimization. Those who are more involved in online interaction are probably at higher risk of being victims of cyberbullying. 

### 4.3. Limitations and Recommendations

#### 4.3.1. Non-Random Sampling

Convenience sampling of schools and students may create sampling bias and weaken the generalizability of the study results. There are three main regions in Hong Kong: Hong Kong Island, Kowloon and the New Territories. No schools on Hong Kong Island were recruited. More prestigious schools are located on Hong Kong Island and, to a lesser extent, in Kowloon [109]. Students in these schools may achieve better academic performance or live in families with higher socioeconomic status. Academic performance may be associated positively with information access online [110] or negatively with smart device addiction [111]. Smart devices may be more affordable among adolescents from wealthier families, although the prices of such devices can be reasonable depending on brand, model and function [112]. Therefore, there may be differences in smart device use and related problems among adolescents recruited from different regions in Hong Kong. 

Schools recruited in this study were assumed to be representative of a majority of registered schools in Hong Kong. The recruited schools were co-educational (co-ed) local non-special schools registered in Hong Kong. They were supposedly following the curriculum and assessment guide proposed by the Hong Kong Education Bureau [40]. Among non-special schools, there were 597 primary schools and 542 secondary schools. Among the primary and secondary schools, the ratios of co-ed to single gender schools were 17.66 and 6.63 respectively. Co-ed local schools were the norm. According to the Education Bureau [113], there were 50 international schools in Hong Kong. Further study could be conducted in international and single gender schools to examine the cultural and gender differences in smart device use and its related health problems.

#### 4.3.2. Respondents’ Availability

The generalizability of the study results to grade 12 students may be lower. Respondents in this study were studying in grades 5 to 12. Around 14% to 20% of them were studying in each of the grades ranging from 7 to 11. However, only around 4% of them were studying in grade 12. In one secondary school, grade 12 students were not available on the day of an arranged school visit. In another, the percentage of grade 12 students was relatively lower. The vice principal stated that this was probably related to overseas study after grade 11. One of the reasons for this is to escape the Diploma of Secondary Education (DSE) examination in grade 12 for undergraduate admission in Hong Kong. 

#### 4.3.3. Invalid Responses and Missing Values

Responses regarding family income in this study may be subject to recall bias. Family income was measured in this study because it was found to have a significant relationship with Internet risk [114]. However, among the responses to family monthly income, some respondents wrote “I guess” or “not sure” beside their choices. As many as 32% of the respondents failed to respond to family monthly income. Among them, some wrote “don’t know” or “can’t tell” instead of choosing an option on the scale. Over 50% of the respondents who responded to the scale measuring income were in the highest four percentiles of Hong Kong family monthly income [66]. This was unlike previous studies in that individuals belonging to high-income groups were found to be less likely to disclose their income [115]. Respondents may want to protect family privacy or may not know their family’s income.

The generalizability of the study results to primary school students may be lower. Of the 1418 respondents who completed and returned their questionnaires, 960 of them proceeded to analysis. Excluded respondents were those who failed to report an age or gender that met the inclusion criteria, or who cited a pattern of smart device use overall that was less than the pattern of any single smart device activity or purpose of device use. Primary school students tended to provide contradictory answers. For instance, some of them responded that the number of days of smart device use overall was “none”, but the number of days on which a smart device was used for messaging was “every day”. Significantly more respondents responded that the overall pattern was less than the pattern of a single activity or a purpose of device use among primary school students (χ^2^ (7, *n* = 1352) = 108.86, *p* < 0.001) and those aged 10–11 (χ^2^ (9, *n* = 1352) = 86.00, *p* < 0.001). Among 960 respondents who proceeded to analysis, only around 13% of them were studying in grades 5 and 6. In future studies, the simplicity, clarity and understandability of the instrument items should be improved. Primary school students may be considered as an individual target group.

Since there was significant difference in the percentage of students being excluded between different education levels, difference in demographics and responses to each item in each grade were examined. Cases with reported ages that did not meet the inclusion criteria were removed. In inferential tests, there were no significant differences in age, gender and family monthly income between included and excluded respondents across grades (Table A1, Table A2 and Table A3). There were significant differences in frequency and time spent on smart device use overall between included and excluded respondents (Table A4 and Table A5). It is probably because, besides respondents who failed to report age or gender that met the inclusion criteria, those who responded that the pattern of smart device use overall was less than the pattern of any smart device activity or purpose of device use were excluded from analysis. However, there is no systematic difference in the purposes, activities or outcomes of smart device use between included and excluded respondents across grades (Table A4, Table A5 and Table A6). The representativeness of the included respondents is acceptable. Yet, related studies may be conducted with larger sample size at each education level in the future.

### 4.4. Implications of Study

Parents, healthcare professionals, teachers and social workers should take measures to protect adolescents against the risks of negative outcomes related to smart device use. A significant number of adolescents perceived the physical and psychosocial outcomes measured in this study. Their patterns of smart device activities put a significant number of them at risk of negative outcomes. Families, teachers and school social workers should be informed about the health risks and benefits of smart device use and the related healthcare resources available. Patterns of smart device activities between adolescents who did and did not perceive the outcomes were different. Therefore, health interventions should be individualized to promote healthy smart device use.

Education should be provided to families on correct posture, taking regular and intermittent rest during smart device use [116,117,118], and avoiding using such devices before going to sleep at night [1,119]. Previous studies found that musculoskeletal problems related to handheld electronic device use were associated with inappropriate postures among children and adolescents [120], and sleep problems were associated with smartphone use before sleep [1].

Guidelines specific to handheld electronic screen-based devices are required. Hong Kong’s Department of Health [10] has made recommendations to parents and teachers regarding general principles in facilitating healthy use of the Internet and electronic screen products among children and adolescents. The principles are being a role model, awareness of consequences, balanced lifestyle, resilience, reasonable rules, communication, trust, and consulting professionals. Their recommendations on healthy use of the Internet and electronic screen products, such as limiting screen time and taking breaks, made reference to international, local and overseas guidelines for computer use at a work station [121,122]. However, smart device use may involve more frequent use, different postures [90], and closer viewing distances [123]. 

Campaigns should be conducted to promote healthy use of smart devices among families. In Hong Kong, the Office for Film, Newspaper and Article Administration (OFNAA) and the Hong Kong Internet Service Providers Association (HKISPA) implement a Code of Practice to co-regulate the publication of obscene and indecent articles on the Internet via Hong Kong-based servers according to the Control of Obscene and Indecent Articles Ordinance (COIAO) [10]. The ordinance may protect children and adolescents against inappropriate content online. However, the regulation by OFNAA and HKISPA relies on complaints on obscene and indecent articles by the public [10]. Therefore, children and adolescents are at risk of being exposed to inappropriate content that has not been subject to complaints or removed. 

## 5. Conclusions

This is the first study to investigate the prevalence and patterns of smart device activities and purposes and perceived outcomes related to smart device use, and the differences in patterns of smart device activities within a specific timeframe between adolescents who did and did not perceive the outcomes. Smart device use for leisure is more common than smart device use for study among Hong Kong adolescents. A significant number of them are probably at risk of negative outcomes related to smart device use. Nearly 60% of the respondents used messaging every day in this study. Nearly 50% browsed information and played games on at least 5 days in a week respectively. Around 50% of them watched TV/movies and nearly 40% posted information on at least 3 days in a week. Nearly 25% of them posted information and around 50% used messaging, browsed information, played games and watched TV/movies for more than 1 h per day respectively. These behavioral patterns were found as the medians among respondents who perceived the outcomes related to smart device use in this study. At least 40% of them perceived physical problems and over 20% perceived family conflict related to smart device use. Evidence on the relationships between patterns of smart device activities and their health impacts among adolescents is limited, and further study is required.

## Figures and Tables

**Table 1:** Demographic characteristics including age, grades and family monthly income, and their gender differences.

Demographics			All		Male		Female		Gender Difference
Age in Years	Range		*n* = 960		*n* = 505		*n* = 455		*t* (*df*) ^1^
			Mean	SD	Mean	SD	Mean	SD	*t* (958) = −0.88, *p* = 0.38
	10–19		13.80	1.93	13.85	1.97	13.74	1.89	
Grade	(Hong Kong System)	(U.S. System)	*n* = 960		*n* = 505		*n* = 455		Chi-Squared ^2^
			*n*	%	*n*	%	*n*	%	χ^2^ (7, *n* = 960) = 6.23, *p* = 0.51
	Primary 5	Grade 5	45	4.7	24	4.8	21	4.6	
	Primary 6	Grade 6	77	8.0	44	8.7	33	7.3	
	Secondary 1	Grade 7	154	16	77	15.2	77	16.9	
	Secondary 2	Grade 8	138	14.4	77	15.2	61	13.4	
	Secondary 3	Grade 9	165	17.2	83	16.4	82	18	
	Secondary 4	Grade 10	196	20.4	110	21.8	86	18.9	
	Secondary 5	Grade 11	146	15.2	67	13.3	79	17.4	
	Secondary 6	Grade 12	39	4.1	23	4.6	16	3.5	
Family Monthly Income	(HKD)	(USD) ^3^	*n* = 650		*n* = 346		*n* = 304		Chi-Squared
			*n*	%	*n*	%	*n*	%	χ^2^ (9, *n* = 650) = 5.00, *p* = 0.83
	1–4000	0.13–516.13	35	5.4	24	6.9	11	3.6	
	4001–8000	516.26–1032.26	27	4.2	15	4.3	12	3.9	
	8001–12,000	1032.39–1548.39	59	9.1	32	9.2	27	8.9	
	12,001–16,000	1548.52–2064.52	67	10.3	35	10.1	32	10.5	
	16,001–20,000	2064.65–2580.65	38	5.8	20	5.8	18	5.9	
	20,001–26,000	2580.77–3354.84	61	9.4	29	8.4	32	10.5	
	26,001–33,000	3354.97–4258.06	72	11.1	41	11.8	31	10.2	
	33,001–43,000	4258.19–5548.39	70	10.8	36	10.4	34	11.2	
	43,001–65,000	5548.52–8387.10	105	16.2	55	15.9	50	16.4	
	Over 65,000	Over 8387.10	116	17.8	59	17.1	57	18.8	

^1^ Independent sample *t* test, 2 tailed. ^2^ Pearson’s chi square test, 2 sided. ^3^ Currency exchange rate at October 2015, rounded up to 2 decimal places.

**Table 2:** Percentages of frequencies and time spent on smart device activities, overall use and its purposes.

	Overall		Leisure		Study		Messaging		Browsing		Gaming		Watching TV/Movie		Posting	
Frequency ^1^	*n* = 960		*n* = 946		*n* = 944		*n* = 953		*n* = 953		*n* = 950		*n* = 954		*n* = 947	
	*n*	%	*n*	%	*n*	%	*n*	%	*n*	%	*n*	%	*n*	%	*n*	%
None	6	0.6	13	1.4	95	10.1	89	9.3	76	8.0	204	21.5	196	20.5	302	31.9
1–2 days	22	2.3	62	6.6	245	26.0	128	13.4	202	21.2	158	16.6	269	28.2	270	28.5
3–4 days	47	4.9	112	11.8	253	26.8	99	10.4	192	20.1	139	14.6	175	18.3	138	14.6
5–6 days	60	6.3	99	10.5	117	12.4	81	8.5	104	10.9	105	11.1	67	7.0	61	6.4
Everyday	825	85.9	660	69.8	234	24.8	556	58.3	379	39.8	344	36.2	247	25.9	176	18.6
Time Spent ^2^	*n* = 959		*n* = 943		*n* = 946		*n* = 950		*n* = 955		*n* = 953		*n* = 957		*n* = 952	
	*n*	%	*n*	%	*n*	%	*n*	%	*n*	%	*n*	%	*n*	%	*n*	%
None	6	0.6	15	1.6	101	10.7	92	9.7	86	9.0	206	21.6	182	19.0	307	32.2
1–60 min	108	11.3	222	23.5	486	51.4	410	43.2	440	46.1	295	31.0	318	33.2	419	44.0
61–120 min	226	23.6	237	25.1	225	23.8	201	21.2	197	20.6	184	19.3	222	23.2	97	10.2
121–180 min	194	20.2	167	17.7	67	7.1	100	10.5	117	12.3	89	9.3	103	10.8	58	6.1
181–240 min	139	14.5	98	10.4	23	2.4	42	4.4	35	3.7	56	5.9	34	3.6	15	1.6
>240 min	286	29.8	204	21.6	44	4.7	105	11.1	80	8.4	123	12.9	98	10.2	56	5.9

^1^ Number of days on which a smart device activity was conducted or smart device was used in the latest week before survey. ^2^ Average time spent on a smart device activity or smart device use per day on which the activity was conducted or the device was used in the latest week before survey.

**Table 3:** Differences in frequencies of smart device activities, overall use and its purposes between primary and secondary school students.

Use	Primary School					Secondary School					Group Difference	
	Frequency ^1^					Frequency						
	No	1–2 Days	3–4 Days	5–6 Days	Every Day	No	1–2 Days	3–4 Days	5–6 Days	Every Day	Mann-Whitney U Test ^2^	*p*
Overall												
*n*	3	13	17	11	78	3	9	30	49	747	*U* = 37,523.5, *z* = −7.86	<0.001
%	2.5	10.7	13.9	9.0	63.9	0.4	1.1	3.6	5.8	89.1		
Study												
*n*	27	47	27	10	8	68	198	226	107	226	*U* = 30,151, *z* = −7.01	<0.001
%	22.7	39.5	22.7	8.4	6.7	8.2	24.0	27.4	13.0	27.4		
Leisure												
*n*	4	25	24	16	51	9	37	88	83	609	*U* = 32,270, *z* = −7.63	<0.001
%	3.3	20.8	20.0	13.3	42.5	1.1	4.5	10.7	10.0	73.7		
Messaging												
*n*	38	37	14	7	24	51	91	85	74	532	*U* = 22,578.5, *z* = −10.89	<0.001
%	31.7	30.8	11.7	5.8	20.0	6.1	10.9	10.2	8.9	63.9		
Browsing												
*n*	43	34	24	6	13	33	168	168	98	366	*U* = 22,485.5, *z* = −10.18	<0.001
%	35.8	28.3	20.0	5.0	10.8	4.0	20.2	20.2	11.8	43.9		
Posting												
*n*	70	27	7	2	14	232	243	131	59	162	*U* = 33,163, *z* = −6.08	<0.001
%	58.3	22.5	5.8	1.7	11.7	28.1	29.4	15.8	7.1	19.6		
Gaming												
*n*	15	34	22	15	36	189	124	117	90	308	*U* = 49,513.5, *z* = −0.36	0.72
%	12.3	27.9	18.0	12.3	29.5	22.8	15.0	14.1	10.9	37.2		
Watching TV/movie												
*n*	23	37	19	8	35	173	232	156	59	212	*U* = 49,510.5, *z* = −0.45	0.65
%	18.9	30.3	15.6	6.6	28.7	20.8	27.9	18.8	7.1	25.5		

^1^ Number of days on which a smart device activity was conducted or smart device was used in the latest week before survey. ^2^ Mann-Whitney U test, 2-tailed.

**Table 4:** Differences in time spent on smart device activities, overall use and its purposes between primary and secondary school students.

Use	Primary School						Secondary School						Group Difference	
	Time Spent ^1^						Time Spent							
	No	1–60 min	61–120 min	121–180 min	181–240 min	>240 min	No	1–60 min	61–120 min	121–180 min	181–240 min	>240 min	Mann-Whitney U Test ^2^	*p*
Overall														
*n*	3	22	39	19	14	25	3	86	187	175	125	261	*U* = 39,892, *z* = −4.01	<0.001
%	2.5	18.0	32.0	15.6	11.5	20.5	0.4	10.3	22.3	20.9	14.9	31.2		
Study														
*n*	30	58	20	5	3	2	71	428	205	62	20	42	*U* = 37,242, *z* = −4.54	<0.001
%	25.4	49.2	16.9	4.2	2.5	1.7	8.6	51.7	24.8	7.5	2.4	5.1		
Leisure														
*n*	5	34	35	17	7	20	10	188	202	150	91	184	*U* = 40,929.5, *z* = −2.87	0.004
%	4.2	28.8	29.7	14.4	5.9	16.9	1.2	22.8	24.5	18.2	11.0	22.3		
Messaging														
*n*	39	52	15	4	3	8	53	358	186	96	39	97	*U* = 31,619, *z* = −6.90	<0.001
%	32.2	43.0	12.4	3.3	2.5	6.6	6.4	43.2	22.4	11.6	4.7	11.7		
Browsing														
*n*	43	55	13	4	0	6	43	385	184	113	35	74	*U* = 28,093, *z* = −8.36	<0.001
%	35.5	45.5	10.7	3.3	0.0	5.0	5.2	46.2	22.1	13.5	4.2	8.9		
Posting														
*n*	65	40	7	2	0	7	242	379	90	56	15	49	*U* = 36,910.5, *z* = −5.04	<0.001
%	53.7	33.1	5.8	1.7	0.0	5.8	29.1	45.6	10.8	6.7	1.8	5.9		
Gaming														
*n*	17	46	32	5	4	18	189	249	152	84	52	105	*U* = 48,933.5, *z* = −0.64	0.53
%	13.9	37.7	26.2	4.1	3.3	14.8	22.7	30.0	18.3	10.1	6.3	12.6		
Watching TV/movie														
*n*	21	49	25	8	5	14	161	269	197	95	29	84	*U* = 49,563, *z* = −0.50	0.62
%	17.2	40.2	20.5	6.6	4.1	11.5	19.3	32.2	23.6	11.4	3.5	10.1		

^1^ Average time spent on a smart device activity or smart device use per day on which the activity was conducted or the device was used in the latest week before survey. ^2^ Mann-Whitney U test, 2-tailed.

**Table 5:** Percentages of frequencies of physical and psychosocial outcomes related to smart device use in a week.

Perceived Outcome	Frequency ^1^									
	None		1–2 Days		3–4 Days		5–6 Days		Everyday	
	*n*	%	*n*	%	*n*	%	*n*	%	*n*	%
Sleep Deprivation	483	50.7	212	22.3	116	12.2	57	6	84	8.8
Eye Discomfort	514	53.9	304	31.9	86	9	20	2.1	29	3
Musculoskeletal Discomfort	597	62.6	225	23.6	80	8.4	20	2.1	32	3.4
Family Conflict	749	78.8	139	14.6	35	3.7	15	1.6	13	1.4
Cyberbullying Victimization	908	95.3	16	1.7	14	1.5	5	.5	10	1

^1^ Number of days on which an outcome related to smart device use was experienced in the latest week before survey.

**Table 6:** Medians and interquartile ranges (IQR) of patterns of smart device activities and pattern differences between adolescents who did and did not perceive the outcomes.

Perceived Outcome		Messaging	Browsing Information	Gaming	Watching TV/Movie	Posting Information
		**Median (IQR) of** **Frequency ^5^**				
Sleep Deprivation	No ^1^	4 (3)	2 (3)	2 (3)	1 (3)	1 (2)
	Yes ^2^	4 (2)	3 (2)	3 (3)	2 (3)	1 (3)
	*U* ^3^	99,250.5 *	95,195 **	101,273	95,013.5 **	101,175
	*Z* ^4^	−3.35	−4.13	−2.44	−4.17	−2.30
Eye Discomfort	No	4 (3)	2 (3)	2 (3)	1 (2)	1 (3)
	Yes	4 (2)	3 (2)	2 (3)	2 (3)	1 (2)
	*U*	108,096.5	103,737	109,028.5	107,614	109,943.5
	*z*	−0.89	−1.90	−0.41	−0.99	0.000
Musculoskeletal Discomfort	No	4 (3)	2 (3)	2 (3)	1 (2)	1 (2)
	Yes	4 (2)	3 (2)	2 (3)	2 (3)	1 (3)
	*U*	95,014.5 *	89,406.5 **	104,437.5	90,790 **	94,202
	*z*	−2.81	−4.00	−0.013	−3.69	−2.50
Family Conflict	No	4 (2)	2 (3)	2 (3)	1 (2)	1 (2)
	Yes	4 (2)	3 (2)	3 (3)	2 (3)	1 (3)
	*U*	67,660.5	66,328	61,997.5 **	63,958 *	65,410.5
	*z*	−2.24	−2.41	−3.85	−3.27	−2.50
Cyberbullying Victimization	No	4 (2)	2 (3)	2 (3)	2 (3)	1 (2)
	Yes	4 (2)	4 (2)	4 (2)	2 (3)	2 (3)
	*U*	17,711	14,619.5 *	15,376 *	15,571 *	13,838.5 **
	*z*	−1.62	−3.09	−2.81	−2.72	−3.68
		**Median (IQR) of** **Time Spent ^6^**				
Sleep Deprivation	No	1 (1)	1 (1)	1 (1)	1 (1)	1 (1)
	Yes	2 (2)	2 (2)	2 (2)	2 (2)	1 (2)
	*U*	95,036.5 **	89,659.5 **	97,849 *	88,732.5 **	100,398.5 *
	*z*	−3.99	−5.65	−3.37	−5.81	−2.80
Eye Discomfort	No	1 (1)	1 (1)	1 (1)	1 (1)	1 (1)
	Yes	2 (2)	2 (2)	2 (2)	2 (2)	1 (2)
	*U*	100,496.5	97,666.5 **	10,2491	100,755.5 *	105,517.5
	*z*	−2.51	−3.52	−2.13	−2.77	−1.37
Musculoskeletal Discomfort	No	1 (1)	1 (1)	1 (2)	1 (1)	1 (1)
	Yes	2 (2)	2 (2)	1.5 (2)	2 (2)	1 (2)
	*U*	87,507.5 **	82,459 **	100,227.5	82,253 **	93,557.5 *
	*z*	−4.36	−5.96	−1.19	−5.92	−2.97
Family Conflict	No	1 (1)	1 (1)	1 (2)	1 (1)	1 (1)
	Yes	2 (2)	2 (2)	2 (3)	2 (2)	1 (2)
	*U*	65,054.5 *	61,226 **	62,147 **	59,728 **	65,556.5
	*z*	−2.71	−4.13	−3.82	−4.52	−2.65
Cyberbullying Victimization	No	1 (2)	1 (1)	1 (2)	1 (1)	1 (1)
	Yes	2 (3)	2 (3)	2 (3)	2 (3)	2 (2)
	*U*	12,741 **	14,014 **	14,327 *	14,242.5*	13,485.5 **
	*z*	−4.40	−3.73	−3.41	−3.30	−4.04

* *p* < 0.01, ** *p* < 0.001 (2-tailed). ^1^ Outcome not perceived in the latest week before survey. ^2^ Outcome perceived in the latest week before survey. ^3^ *U*-value of Mann-Whitney U test. ^4^ *z*-value of Mann-Whitney U test. ^5^ Number of days on which a smart device activity was conducted in the latest week before survey, where 0 = “None”, 1 = “1–2 days”, 2 = “3–4 days”, 3 = “5–6 days”, 4 = “Everyday”. ^6^ Average time spent on a smart device activity per day on which the activity was conducted in the latest week before survey, where 0 = “None”, 1 = “1–60 min”, 2 = “61–120 min”, 3 = “121–180 min”, 4 = “181–240 min”, 5 = “Over 240 min”.

**Table 7:** Complex sample binary logistic regression estimating likelihood of perceived outcomes among primary school students.

Model	B ^1^	*SE* ^2^	χ^2 3^	*df* ^4^	*p* ^5^	*OR* ^6^	95% CI ^7^		Nagelkerke R^2^	C.A. ^10^
							*LL* ^8^	*UL* ^9^		
Eye Discomfort									0.07	64.8
Frequency of Study	−0.43	0.13	11.09	1	0.001	0.65	0.48	0.88		
Musculoskeletal Discomfort									0.15	79.7
Time Spent on Gaming	−0.74	0.18	17.12	1	<0.001	0.48	0.32	0.72		
Sleep Deprivation									0.11	73.7
Overall Time Spent	0.27	0.16	2.77	1	0.096	1.31	0.90	1.89		
Time Spent on Gaming	0.23	0.29	0.62	1	0.43	1.25	0.65	2.42		
Frequency of Gaming	−0.06	0.20	0.10	1	0.75	0.94	0.60	1.48		
Family Conflict									0.48	90.1
Overall Time Spent	1.22	0.50	5.93	1	0.02	3.40	1.07	10.82		
Frequency of Study	−1.00	0.49	4.28	1	0.04	0.37	0.12	1.12		
Time Spent on Watching TV/movie	0.18	0.20	0.83	1	0.36	1.20	0.76	1.89		

^1^ Log-odds. ^2^ Standard error of B value. ^3^ Adjusted Wald chi-squared. ^4^ Degrees of freedom. ^5^ *p*-value adjusted with sequential Bonferroni method. ^6^ Odds ratio. ^7^ 95% confidence interval. ^8^ Lower limit if 95% CI. ^9^ Upper limit of 95% CI. ^10^ Classification accuracy in percentage.

**Table 8:** Complex sample binary logistic regression estimating likelihood of perceived outcomes among secondary school students.

Model	B ^1^	*SE* ^2^	χ^2 3^	*df* ^4^	*p* ^5^	*OR* ^6^	95% CI ^7^		Nagelkerke R^2^	C.A. ^10^
							*LL* ^8^	*UL* ^9^		
Eye Discomfort									0.03	57.1
Time Spent on Messaging	−0.13	0.04	9.33	1	0.002	0.88	0.80	0.97		
Time Spent on Browsing	0.18	0.06	8.10	1	0.004	1.20	1.04	1.38		
Time Spent on Watching TV/movie	0.09	0.03	6.27	1	0.01	1.09	1.01	1.18		
Time Spent on Leisure	0.07	0.06	1.52	1	0.22	1.08	0.94	1.23		
Time Spent on Gaming	−0.01	0.05	0.02	1	0.89	0.99	0.88	1.12		
Musculoskeletal Discomfort									0.11	64.0
Time Spent on Browsing	0.31	0.09	12.75	1	<0.001	1.36	1.12	1.64		
Time Spent on Watching TV/movie	0.25	0.04	36.39	1	<0.001	1.29	1.17	1.41		
Time Spent on Gaming	−0.19	0.06	11.37	1	0.001	0.83	0.73	0.94		
Overall Time Spent	0.14	0.07	4.53	1	0.03	1.15	0.99	1.34		
Time Spent on Posting	−0.12	0.07	3.05	1	0.08	0.89	0.77	1.03		
Overall Frequency	0.26	0.25	1.06	1	0.30	1.30	0.74	2.28		
Frequency of Browsing	−0.04	0.06	0.48	1	0.49	0.96	0.83	1.10		
Time Spent on Messaging	−0.04	0.08	0.26	1	0.61	0.96	0.80	1.15		
Frequency of Leisure	0.02	0.09	0.06	1	0.80	1.02	0.83	1.26		
Frequency of Messaging	0.02	0.09	0.05	1	0.82	1.02	0.83	1.26		
Time Spent on Study	−0.001	0.07	0.00	1	0.99	1.00	0.86	1.17		
Sleep Deprivation									0.14	65.4
Age	0.20	0.04	22.17	1	<0.001	1.23	1.11	1.35		
Time Spent on Watching TV/movie	0.25	0.06	19.70	1	<0.001	1.28	1.13	1.46		
Frequency of Leisure	0.26	0.11	5.25	1	0.02	1.29	1.01	1.66		
Overall Time Spent	0.13	0.06	5.06	1	0.02	1.14	1.00	1.29		
Time Spent on Gaming	−0.09	0.06	2.60	1	0.11	0.91	0.80	1.04		
Time Spent on Posting	−0.12	0.08	2.24	1	0.13	0.89	0.75	1.06		
Time Spent on Messaging	−0.07	0.05	2.09	1	0.15	0.94	0.84	1.04		
Time Spent on Browsing	0.11	0.09	1.34	1	0.25	1.12	0.91	1.37		
Frequency of Messaging	−0.01	0.06	0.02	1	0.89	0.99	0.88	1.12		
Frequency of Browsing	−0.002	0.03	0.01	1	0.94	1.00	0.93	1.07		
Overall Frequency	0.01	0.14	0.001	1	0.97	1.01	0.73	1.38		
Family Conflict									0.07	78.6
Time Spent on Watching TV/movie	0.22	0.06	12.47	1	<0.001	1.24	1.08	1.43		
Frequency of Leisure	0.27	0.09	8.37	1	0.004	1.31	1.06	1.61		
Time Spent on Messaging	−0.16	0.06	7.35	1	0.01	0.85	0.75	0.97		
Time Spent on Browsing	0.20	0.09	4.99	1	0.03	1.22	1.00	1.50		
Time Spent on Gaming	−0.09	0.05	2.79	1	0.10	0.91	0.81	1.03		
Frequency of Browsing	−0.05	0.05	1.18	1	0.28	0.95	0.85	1.06		
Time Spent on Posting	0.08	0.10	0.74	1	0.39	1.09	0.88	1.34		
Time Spent on Leisure	0.04	0.05	0.68	1	0.41	1.04	0.93	1.18		
Cyberbullying Victimization									0.35	98.6
Time Spent on Posting	0.89	0.44	4.07	1	0.04	2.43	0.91	6.48		
Time Spent on Gaming	0.43	0.34	1.63	1	0.20	1.54	0.72	3.29		
Frequency of Watching TV/movie	0.14	0.14	1.01	1	0.32	1.15	0.85	1.56		
Time Spent on Leisure	−0.17	0.25	0.47	1	0.49	0.84	0.48	1.48		
Time Spent on Messaging	−0.04	0.18	0.06	1	0.81	0.96	0.65	1.42		
Time Spent on Browsing	−0.01	0.28	0.002	1	0.96	0.99	0.53	1.84		

^1^ Log-odds. ^2^ Standard error of B value. ^3^ Adjusted Wald chi-squared. ^4^ Degrees of freedom. ^5^ *p*-value adjusted with sequential Bonferroni method. ^6^ Odds ratio. ^7^ 95% confidence interval. ^8^ Lower limit if 95% CI. ^9^ Upper limit of 95% CI. ^10^ Classification accuracy in percentage.

## Appendix A

Questionnaire

Smart devices include smartphone and tablet computer.

Please choose an answer for each item, and put a tick inside the box if appropriate.

Number of days on which smart device was used in the last week					
	None	1–2 days	3–4 days	5–6 days	Everyday
1. Overall	□	□	□	□	□
2. For study	□	□	□	□	□
3. For leisure	□	□	□	□	□

Average time spent on smart device use per day on which the device was used in the last week						
	None	1–60 min	61–120 min	121–180 min	181–240 min	Over 240 min
4. Overall	□	□	□	□	□	□
5. For study	□	□	□	□	□	□
6. For leisure	□	□	□	□	□	□

Number of days on which the following smart device activity was conducted in the last week					
	None	1–2 days	3–4 days	5–6 days	Everyday
7. Watching TV/Movie	□	□	□	□	□
8. Browsing Information	□	□	□	□	□
9. Posting Information	□	□	□	□	□
10. Messaging	□	□	□	□	□
11. Gaming	□	□	□	□	□

Average time spent on the following smart device activity per day on which the activity was conducted in the last week						
	None	1–60 min	61–120 min	121–180 min	181–240 min	Over 240 min
12. Watching TV/Movie	□	□	□	□	□	□
13. Browsing Information	□	□	□	□	□	□
14. Posting Information	□	□	□	□	□	□
15. Messaging	□	□	□	□	□	□
16. Gaming	□	□	□	□	□	□

Number of days on which the followings were experienced relating to smart device use in the last week					
	None	1–2 days	3–4 days	5–6 days	Everyday
17. Eye discomfort	□	□	□	□	□
18. Musculoskeletal discomfort	□	□	□	□	□
19. Sleep deprivation	□	□	□	□	□
20. Family conflict	□	□	□	□	□
21. Cyberbullying victimization	□	□	□	□	□

22. Age: __________
23. Gender: □Male, □Female
24. Education Level: □ Primary 4, □ Primary 5, □ Primary 6, □ Secondary 1, □ Secondary 2, □ Secondary 3, □ Secondary 4, □ Secondary 5, □ Secondary 6
25. Family monthly income: □ HKD 1–4000, □ HKD 4001–8000, □ HKD 8001–12,000, □ HKD 12,001–16,000, □ HKD 16,001–20,000, □ HKD 20,001–26,000, □ HKD 26,001–33,000, □ HKD 33,001–43,000, □ HKD 43,001–65,000, □ Over HKD 65,000

Date: ____________________ Class: ___________________ Class number: _________________

## Appendix B

**Table A1:** Difference in age between included and excluded respondents by grades.

Grade	Included		Excluded		Group Difference	
	Mean	SD	Mean	SD	*t* (*df*) ^1^	*p*
5	10.31	0.60	10.48	0.60	*t* (112) = −1.25	0.22
6	11.22	0.79	11.11	0.69	*t* (145) = 0.87	0.39
7	11.94	0.53	11.98	0.71	*t* (210) = −0.46	0.65
8	12.99	0.60	13.12	0.88	*t* (69.6) = −0.93	0.36
9	14.05	0.56	14.19	0.68	*t* (64.98) = −1.27	0.21
10	15.04	0.56	15.00	0.68	*t* (242) = 0.43	0.67
11	16.11	0.78	15.93	0.64	*t* (176) = 1.15	0.25
12	17.08	0.70	17.40	0.74	*t* (52) = −1.49	0.14

^1^ Independent sample *t* test, 2 tailed.

**Table A2:** Difference in gender between included and excluded respondents by grades.

Grade	Included		Excluded		Group Difference	
	Male	Female	Male	Female		
	*n*	*n*	*n*	*n*	Chi-Squared ^1^	*p*
5	24	21	40	28	χ^2^ (1, *n* = 113) = 0.15	0.70
6	45	33	41	30	χ^2^ (1, *n* = 149) = 0.0	1.0
7	77	77	33	25	χ^2^ (1, *n* = 212) = 0.55	0.46
8	79	64	30	22	χ^2^ (1, *n* = 195) = 0.02	0.89
9	87	83	28	18	χ^2^ (1, *n* = 216) = 1.01	0.32
10	111	86	24	24	χ^2^ (1, *n* = 245) = 0.40	0.53
11	72	81	13	17	χ^2^ (1, *n* = 183) = 0.03	0.86
12	25	16	11	4	χ^2^ (1, *n* = 56) = 0.29	0.59

^1^ Pearson’s chi square test, 2-sided.

**Table A3:** Difference in family monthly income percentile between included and excluded respondents by grades.

Grade		Family Monthly Income Percentile										Group Difference	
		1st	2nd	3rd	4th	5th	6th	7th	8th	9th	10th		
		*n*	*n*	*n*	*n*	*n*	*n*	*n*	*n*	*n*	*n*	Chi-Squared ^1^	*p*
5	Included	5	3	9	7	1	2	1	2	3	5	χ^2^ (9, *n* = 92) = 7.16	0.62
	Excluded	7	8	14	9	3	2	3	4	0	4		
6	Included	4	10	13	12	8	8	7	2	4	3	χ^2^ (9, *n* = 128) = 12.92	0.17
	Excluded	8	9	14	9	5	5	2	5	0	0		
7	Included	6	2	4	9	5	8	9	21	25	24	χ^2^ (9, *n* = 160) = 9.16	0.42
	Excluded	0	4	3	5	2	5	2	7	11	8		
8	Included	5	3	5	7	7	6	11	8	20	15	χ^2^ (9, *n* = 112) = 8.30	0.50
	Excluded	0	1	5	3	1	1	3	3	3	5		
9	Included	9	3	3	8	5	8	10	7	9	11	χ^2^ (9, *n* = 103) = 8.49	0.49
	Excluded	4	1	2	6	1	5	0	4	5	2		
10	Included	1	0	13	10	7	16	18	18	26	44	χ^2^ (8, *n* = 183) = 10.40	0.24
	Excluded	1	0	1	6	2	5	3	3	3	6		
11	Included	2	1	8	9	6	11	15	9	18	13	χ^2^ (9, *n* = 108) = 10.31	0.33
	Excluded	2	0	0	2	1	1	2	4	1	3		
12	Included	5	5	4	7	0	3	3	4	0	2	χ^2^ (8, *n* = 47) = 9.48	0.30
	Excluded	0	0	2	3	1	2	3	3	0	0		

^1^ Pearson’s chi square test, 2-sided.

**Table A4:** Differences in frequencies of smart device activities, overall use and its purposes between included and excluded respondents by grades.

Grade	Included					Excluded					Group Difference	
	Frequency ^1^					Frequency						
	No	1–2 Days	3–4 Days	5–6 Days	Every Day	No	1–2 Days	3–4 Days	5–6 Days	Every Day		
	*n*	*n*	*n*	*n*	*n*	*n*	*n*	*n*	*n*	*n*	Chi-Squared ^2^	*p*
	Overall					Overall						
5	1	9	7	1	27	18	14	9	5	18	χ^2^ (4, *n* = 109) = 18.26	0.001
6	2	4	10	10	54	22	11	13	6	10	χ^2^ (4, *n* = 142) = 50.10	<0.001
7	1	3	12	23	115	6	10	6	10	25	χ^2^ (4, *n* = 211) = 35.16	<0.001
8	1	1	6	10	126	3	14	10	8	15	χ^2^ (4, *n* = 194) = 72.30	<0.001
9	0	2	6	9	155	8	3	7	4	21	χ^2^ (4, *n* = 215) = 54.41	<0.001
10	0	1	5	5	187	3	1	3	1	40	χ^2^ (4, *n* = 246) = 15.76	0.003
11	1	2	0	4	159	3	0	3	2	22	χ^2^ (4, *n* = 196) = 30.85	<0.001
12	0	0	1	0	40	1	0	1	2	11	χ^2^ (3, *n* = 56) = 9.46	0.024
	Study					Study						
5	10	14	9	8	3	17	15	8	9	13	χ^2^ (4, *n* = 106) = 5.31	0.26
6	18	33	19	3	5	18	26	11	3	7	χ^2^ (4, *n* = 143) = 2.13	0.71
7	9	41	44	23	33	5	13	19	6	15	χ^2^ (4, *n* = 208) = 2.00	0.74
8	12	32	34	21	43	2	16	18	7	9	χ^2^ (4, *n* = 194) = 6.22	0.18
9	15	32	59	29	36	8	16	3	7	9	χ^2^ (4, *n* = 214) = 17.33	0.002
10	22	48	46	20	58	5	9	14	4	17	χ^2^ (4, *n* = 243) = 1.57	0.81
11	9	43	48	18	46	4	6	9	5	6	χ^2^ (4, *n* = 194) = 4.03	0.40
12	4	11	6	0	20	0	3	7	1	4	χ^2^ (4, *n* = 56) = 10.51	0.03
	Leisure					Leisure						
5	2	12	7	7	17	5	21	8	8	24	χ^2^ (4, *n* = 111) = 1.14	0.89
6	2	14	17	9	36	5	10	19	7	28	χ^2^ (4, *n* = 147) = 2.77	0.60
7	4	17	25	20	85	4	8	6	12	29	χ^2^ (4, *n* = 210) = 5.06	0.28
8	2	6	19	17	98	2	4	11	9	27	χ^2^ (4, *n* = 195) = 5.94	0.20
9	1	9	19	17	125	1	5	5	3	31	χ^2^ (4, *n* = 216) = 3.41	0.49
10	0	4	13	15	162	2	2	5	3	37	χ^2^ (4, *n* = 243) = 9.66	0.05
11	2	3	13	11	135	1	1	4	3	22	χ^2^ (4, *n* = 195) = 2.41	0.66
12	0	1	3	4	33	0	0	0	4	11	χ^2^ (3, *n* = 56) = 3.73	0.29
	Watching TV/movie					Watching TV/movie						
5	7	17	5	2	14	16	19	6	5	22	χ^2^ (4, *n* = 113) = 2.20	0.70
6	16	20	15	6	23	6	14	15	5	28	χ^2^ (4, *n* = 148) = 5.25	0.26
7	32	48	29	13	31	13	15	11	6	13	χ^2^ (4, *n* = 211) = 0.73	0.95
8	34	36	23	7	43	10	10	11	5	17	χ^2^ (4, *n* = 196) = 2.91	0.57
9	34	50	37	12	39	10	16	3	3	14	χ^2^ (4, *n* = 218) = 5.82	0.21
10	43	49	31	14	58	7	14	11	3	14	χ^2^ (4, *n* = 244) = 2.39	0.66
11	34	53	33	12	33	8	10	5	1	6	χ^2^ (4, *n* = 195) = 1.18	0.88
12	9	6	7	3	16	3	5	3	1	2	χ^2^ (4, *n* = 55) = 4.38	0.36
	Browsing					Browsing						
5	13	11	10	4	5	29	16	9	4	8	χ^2^ (4, *n* = 109) = 3.05	0.55
6	30	23	14	2	11	11	30	13	4	10	χ^2^ (4, *n* = 148) = 9.57	0.05
7	14	53	41	15	29	4	16	18	5	14	χ^2^ (4, *n* = 209) = 1.78	0.78
8	8	29	30	15	61	4	13	16	10	9	χ^2^ (4, *n* = 195) = 11.32	0.02
9	4	28	34	26	80	4	7	7	7	22	χ^2^ (4, *n* = 219) = 4.38	0.36
10	3	32	35	29	99	3	7	15	7	17	χ^2^ (4, *n* = 247) = 8.73	0.07
11	2	27	32	11	92	2	4	4	5	15	χ^2^ (4, *n* = 194) = 7.55	0.11
12	2	3	4	4	28	1	2	3	2	6	χ^2^ (4, *n* = 55) = 3.06	0.55
	Posting					Posting						
5	22	9	4	2	6	39	12	5	8	2	χ^2^ (4, *n* = 109) = 6.31	0.18
6	48	18	3	0	10	31	21	9	3	6	χ^2^ (4, *n* = 149) = 10.38	0.03
7	51	52	25	5	18	20	18	8	4	8	χ^2^ (4, *n* = 209) = 1.72	0.79
8	37	33	26	7	39	12	16	10	3	10	χ^2^ (4, *n* = 193) = 2.08	0.72
9	50	48	28	15	31	12	19	4	2	9	χ^2^ (4, *n* = 218) = 4.59	0.33
10	42	53	32	24	44	16	9	13	4	7	χ^2^ (4, *n* = 244) = 7.28	0.12
11	55	61	21	5	22	9	7	5	2	7	χ^2^ (4, *n* = 194) = 4.36	0.36
12	10	8	5	3	14	6	2	3	1	2	χ^2^ (4, *n* = 54) = 3.36	0.50
	Messaging					Messaging						
5	12	14	4	5	9	21	17	9	6	12	χ^2^ (4, *n* = 109) = 1.19	0.88
6	26	23	10	2	17	15	13	13	7	21	χ^2^ (4, *n* = 147) = 8.80	0.07
7	17	30	22	17	66	8	6	5	4	35	χ^2^ (4, *n* = 210) = 6.79	0.15
8	13	14	15	10	91	4	11	4	7	26	χ^2^ (4, *n* = 195) = 7.25	0.12
9	6	18	24	18	106	4	10	3	2	28	χ^2^ (4, *n* = 219) = 8.71	0.07
10	9	17	13	20	139	4	2	2	3	38	χ^2^ (4, *n* = 247) = 3.38	0.50
11	4	13	11	7	129	2	1	4	3	20	χ^2^ (4, *n* = 194) = 5.74	0.22
12	2	2	5	3	29	1	2	1	1	9	χ^2^ (4, *n* = 55) = 1.68	0.80
	Gaming					Gaming						
5	3	12	9	6	15	8	21	15	3	21	χ^2^ (4, *n* = 113) = 3.70	0.45
6	12	23	13	9	23	9	12	15	6	28	χ^2^ (4, *n* = 150) = 4.47	0.35
7	37	27	26	16	43	9	11	12	9	18	χ^2^ (4, *n* = 208) = 2.71	0.61
8	35	18	22	9	59	7	13	7	7	20	χ^2^ (4, *n* = 197) = 8.20	0.09
9	40	33	20	19	60	9	7	10	6	15	χ^2^ (4, *n* = 219) = 3.35	0.50
10	39	22	23	25	87	12	6	5	4	22	χ^2^ (4, *n* = 245) = 1.21	0.88
11	47	26	27	18	46	7	7	4	5	7	χ^2^ (4, *n* = 194) = 2.15	0.71
12	4	4	3	5	25	5	2	2	2	3	χ^2^ (4, *n* = 55) = 8.29	0.08

^1^ Number of days on which a smart device activity was conducted or smart device was used in the latest week before survey. ^2^ Pearson’s chi square test, 2-sided.

**Table A5:** Differences in time spent on smart device activities, overall use and its purposes between included and excluded respondents by grades.

Grade	Included						Excluded						Group Difference	
	Time Spent ^1^						Time Spent							
	No	1–60 min	61–120 min	121–180 min	181–240 min	>240 min	No	1–60 min	61–120 min	121–180 min	181–240 min	>240 min		
	*n*	*n*	*n*	*n*	*n*	*n*	*n*	*n*	*n*	*n*	*n*	*n*	Chi-Squared ^2^	*p*
	Overall						Overall							
5	1	9	12	7	8	8	17	21	10	8	2	2	χ^2^ (5, *n* = 105) = 24.84 <0.001	
6	2	13	28	13	6	18	18	17	12	5	5	5	χ^2^ (5, *n* = 142) = 28.91 <0.001	
7	1	35	55	27	12	24	5	18	14	14	5	0	χ^2^ (5, *n* = 210) = 22.70 <0.001	
8	1	18	39	29	23	34	4	19	15	8	3	2	χ^2^ (5, *n* = 195) = 30.92 <0.001	
9	0	15	35	43	27	52	5	10	12	10	4	0	χ^2^ (5, *n* = 213) = 42.30 <0.001	
10	0	15	37	40	31	75	2	7	15	19	5	0	χ^2^ (5, *n* = 246) = 38.21 <0.001	
11	1	5	31	37	34	57	4	2	14	5	4	1	χ^2^ (5, *n* = 195) = 34.92 <0.001	
12	0	1	3	8	4	25	0	2	5	6	2	0	χ^2^ (4, *n* = 56) = 18.76	0.001
	Study						Study							
5	9	21	10	3	0	2	16	19	15	5	4	1	χ^2^ (5, *n* = 105) = 5.87	0.32
6	22	38	10	3	3	0	21	27	7	6	1	1	χ^2^ (5, *n* = 139) = 4.24	0.52
7	12	96	31	6	0	5	5	26	21	4	0	1	χ^2^ (4, *n* = 207) = 7.84	0.10
8	12	73	36	18	2	3	5	28	12	2	4	1	χ^2^ (5, *n* = 196) = 7.97	0.16
9	14	84	50	10	7	6	10	21	10	1	3	0	χ^2^ (5, *n* = 216) = 10.00	0.08
10	23	96	45	15	5	11	6	22	10	10	1	0	χ^2^ (5, *n* = 244) = 9.27	0.10
11	10	85	42	15	5	7	3	18	7	2	0	0	χ^2^ (5, *n* = 194) = 3.32	0.65
12	2	16	7	3	1	12	0	11	3	0	0	1	χ^2^ (5, *n* = 56) = 7.35	0.20
	Leisure						Leisure							
5	2	16	11	7	3	6	7	29	11	10	2	7	χ^2^ (5, *n* = 111) = 3.49	0.63
6	3	19	24	11	4	15	4	22	13	12	11	9	χ^2^ (5, *n* = 147) = 8.28	0.14
7	5	66	40	16	5	18	2	23	11	10	9	2	χ^2^ (5, *n* = 207) = 15.16	0.01
8	2	41	33	29	13	26	2	18	12	9	5	8	χ^2^ (5, *n* = 198) = 1.85	0.87
9	1	39	44	32	20	34	2	11	12	8	8	5	χ^2^ (5, *n* = 216) = 6.30	0.28
10	0	25	52	34	26	56	2	11	11	15	6	4	χ^2^ (5, n = 242) = 20.71	0.001
11	2	22	41	40	25	34	2	9	10	5	2	3	χ^2^ (5, *n* = 195) = 11.91	0.04
12	0	4	5	8	5	19	0	4	3	7	1	0	χ^2^ (4, *n* = 56) = 12.95	0.01
	Watching TV/movie						Watching TV/movie							
5	8	17	11	1	3	5	16	20	14	10	2	6	χ^2^ (5, *n* = 113) = 6.51	0.26
6	13	32	15	8	2	10	7	13	19	16	6	8	χ^2^ (5, *n* = 149) = 14.45	0.01
7	35	64	30	12	4	9	12	17	16	8	2	3	χ^2^ (5, *n* = 212) = 4.76	0.45
8	25	53	35	14	6	11	12	13	10	13	1	4	χ^2^ (5, *n* = 197) = 9.58	0.09
9	33	57	41	21	4	16	9	13	11	7	3	4	χ^2^ (5, *n* = 219) = 2.48	0.78
10	41	61	37	23	7	27	7	5	17	9	4	7	χ^2^ (5, *n* = 245) = 14.87	0.01
11	28	42	50	21	6	18	7	10	6	6	1	1	χ^2^ (5, *n* = 196) = 4.44	0.49
12	9	1	13	8	3	7	2	3	6	3	0	1	χ^2^ (5, *n* = 56) = 7.31	0.20
	Browsing						Browsing							
5	15	18	7	2	0	2	30	24	7	2	1	1	χ^2^ (5, *n* = 109) = 3.27	0.66
6	28	39	6	3	0	4	16	29	10	6	5	2	χ^2^ (5, *n* = 148) = 11.51	0.04
7	19	93	26	10	3	3	4	26	18	5	2	3	χ^2^ (5, *n* = 212) = 9.25	0.10
8	11	69	33	14	5	11	7	25	11	6	2	2	χ^2^ (5, *n* = 196) = 2.38	0.80
9	5	88	33	25	10	11	3	19	9	7	6	2	χ^2^ (5, *n* = 218) = 4.87	0.43
10	5	83	45	33	6	24	2	13	14	10	3	7	χ^2^ (5, *n* = 245) = 4.75	0.45
11	3	58	48	27	8	21	2	8	4	10	4	2	χ^2^ (5, *n* = 195) = 12.84	0.03
12	0	9	10	9	4	9	1	3	6	2	2	1	χ^2^ (5, *n* = 56) = 5.67	0.34
	Posting						Posting							
5	22	14	5	0	0	3	40	12	9	4	0	1	χ^2^ (4, *n* = 110) = 7.42	0.12
6	43	27	2	3	0	4	30	19	9	6	2	1	χ^2^ (5, *n* = 146) = 12.06	0.03
7	54	77	14	5	0	3	17	22	13	2	1	2	χ^2^ (5, *n* = 210) = 10.71	0.06
8	38	65	20	12	0	7	14	26	6	3	1	3	χ^2^ (5, *n* = 195) = 3.42	0.64
9	49	84	19	11	4	5	9	23	8	4	2	0	χ^2^ (5, *n* = 218) = 4.43	0.49
10	46	82	24	13	9	21	16	14	9	5	2	3	χ^2^ (5, *n* = 244) = 5.69	0.34
11	54	74	16	9	0	12	10	7	5	5	2	1	χ^2^ (5, *n* = 195) = 20.01	0.001
12	12	14	2	6	2	5	3	7	2	1	1	1	χ^2^ (5, *n* = 56) = 2.85	0.72
	Messaging						Messaging							
5	14	16	7	2	2	3	28	21	7	4	2	4	χ^2^ (5, *n* = 110) = 1.83	0.87
6	26	36	8	3	1	5	16	24	11	5	3	9	χ^2^ (5, *n* = 147) = 7.11	0.21
7	19	77	30	14	6	7	6	20	14	6	5	6	χ^2^ (5, *n* = 210) = 7.02	0.22
8	14	66	31	13	6	13	4	17	14	7	5	6	χ^2^ (5, *n* = 196) = 5.13	0.40
9	6	83	45	17	7	13	4	16	11	5	7	3	χ^2^ (5, *n* = 217) = 10.74	0.06
10	8	79	45	21	9	32	4	9	8	5	12	11	χ^2^ (5, *n* = 243) = 26.17 <0.001	
11	4	57	40	24	10	29	2	4	4	10	5	5	χ^2^ (5, *n* = 194) = 15.27	0.01
12	2	13	6	10	2	8	0	2	4	3	3	2	χ^2^ (5, *n* = 55) = 6.30	0.28
	Gaming						Gaming							
5	3	21	12	1	3	5	10	31	13	2	7	6	χ^2^ (5, *n* = 114) = 2.83	0.73
6	14	26	20	5	1	14	9	16	15	9	10	10	χ^2^ (5, *n* = 149) = 12.61	0.03
7	42	55	28	11	8	9	9	21	11	4	10	4	χ^2^ (5, *n* = 212) = 9.59	0.09
8	33	42	29	12	7	20	7	19	8	11	1	8	χ^2^ (5, *n* = 197) = 8.76	0.12
9	38	58	27	21	6	20	7	10	11	12	5	1	χ^2^ (5, *n* = 216) = 15.70	0.01
10	40	53	38	18	19	29	12	12	5	9	4	7	χ^2^ (5, *n* = 246) = 5.41	0.37
11	45	47	27	17	8	20	8	10	4	6	2	1	χ^2^ (5, *n* = 195) = 4.21	0.52
12	4	4	7	9	6	11	4	5	1	3	2	0	χ^2^ (5, *n* = 56) = 10.89	0.05

^1^ Average time spent on a smart device activity or smart device use per day on which the activity was conducted or the device was used in the latest week before survey. ^2^ Pearson’s chi square test, 2-sided.

**Table A6:** Differences in frequencies of physical and psychosocial outcomes related to smart device use in a week between included and excluded respondents by grades.

Grade	Included					Excluded					Group Difference	
	Frequency ^1^					Frequency						
	No	1–2 Days	3–4 Days	5–6 Days	Every Day	No	1–2 Days	3–4 Days	5–6 Days	Every Day	Mann-Whitney U Test ^2^	*p*
	*n*	*n*	*n*	*n*	*n*	*n*	*n*	*n*	*n*	*n*		
	Eye Discomfort					Eye Discomfort						
5	30	10	3	0	2	47	15	3	3	0	*U* = 1483.5, *z* = −0.33	0.74
6	48	23	5	1	3	45	20	4	2	0	*U* = 2717, *z* = −0.53	0.60
7	102	44	3	1	4	36	12	4	2	3	*U* = 4109, *z* = −0.85	0.40
8	83	42	12	1	2	25	15	8	2	2	*U* = 3081.5, *z* = -1.83	0.07
9	80	58	22	3	7	26	13	4	4	0	*U* = 3699, *z* = −0.84	0.40
10	90	72	20	8	5	18	19	5	2	4	*U* = 4154, *z* = −1.30	0.19
11	81	54	19	6	6	16	8	5	2	0	*U* = 2548.5, *z* = −0.09	0.93
12	20	12	5	0	4	5	7	3	0	0	*U* = 279.5, *z* = −0.56	0.58
	Musculoskeletal Discomfort					Musculoskeletal Discomfort						
5	37	7	0	0	1	52	13	3	0	0	*U* = 1439.5, *z* = −0.75	0.46
6	58	13	2	1	5	49	15	2	2	3	*U* = 2700, *z* = −0.50	0.62
7	110	34	3	2	5	44	10	2	0	2	*U* = 4284, *z* = −0.59	0.56
8	98	29	9	4	1	32	8	9	0	3	*U* = 3259, *z* = −1.43	0.15
9	105	41	15	4	6	24	13	7	2	1	*U* = 3577, *z* = −1.31	0.19
10	98	56	28	4	9	24	15	4	2	3	*U* = 4677.5, *z* = −0.01	10.00
11	92	47	17	3	7	14	10	5	2	0	*U* = 2294.5, *z* = −1.06	0.29
12	24	8	6	2	1	4	6	4	1	0	*U* = 220, *z* = −1.75	0.08
	Sleep Deprivation					Sleep Deprivation						
5	33	6	0	2	4	46	14	2	2	4	*U* = 1467, *z* = −0.46	0.65
6	50	14	5	3	7	48	12	5	3	3	*U* = 2654.5, *z* = −0.67	0.50
7	100	27	12	4	11	38	7	5	3	5	*U* = 4407, *z* = −0.18	0.86
8	86	25	20	4	6	30	11	3	4	4	*U* = 3511.5, *z* = −0.51	0.61
9	88	35	21	9	18	23	7	10	4	3	*U* = 3871.5, *z* = −0.42	0.68
10	72	51	33	12	26	18	16	2	2	10	*U* = 4621, *z* = −0.08	0.93
11	58	46	28	19	14	13	6	5	4	3	*U* = 2493, *z* = −0.23	0.82
12	13	13	4	5	6	5	5	1	0	4	*U* = 305, *z* = −0.05	0.96
	Family Conflict					Family Conflict						
5	37	6	0	0	2	60	5	3	0	0	*U* = 1439, *z* = −0.88	0.38
6	69	5	4	0	2	63	6	1	1	0	*U* = 2756.5, *z* = −0.54	0.59
7	131	17	6	0	0	51	6	1	0	0	*U* = 4328.5, *z* = −0.57	0.57
8	111	18	7	1	3	36	13	1	0	2	*U* = 3312.5, *z* = -1.29	0.20
9	131	33	2	4	1	31	7	5	0	4	*U* = 3467, *z* = −1.89	0.06
10	145	32	11	6	1	29	9	6	1	3	*U* = 3939, *z* = −2.14	0.03
11	121	27	7	4	5	23	3	2	0	3	*U* = 2501.5, *z* = −0.18	0.86
12	31	5	1	1	2	11	3	0	1	0	*U* = 291.5, *z* = −0.22	0.83
	Cyberbullying Victimization					Cyberbullying Victimization						
5	42	1	0	0	2	66	2	0	0	0	*U* = 1471, *z* = −0.97	0.33
6	78	0	0	0	1	68	1	0	2	0	*U* = 2723, *z* = −1.10	0.27
7	151	2	0	0	0	56	2	0	0	0	*U* = 4342, *z* = −1.02	0.31
8	135	2	3	0	1	49	1	1	0	1	*U* = 3609.5, *z* = −0.45	0.65
9	165	1	1	1	3	43	0	2	0	2	*U* = 3817.5, *z* = −1.45	0.15
10	184	3	5	1	2	44	3	1	0	0	*U* = 4566, *z* = −0.63	0.53
11	151	5	5	3	2	28	0	2	0	1	*U* = 2550, *z* = −0.16	0.88
12	38	2	0	0	1	12	2	1	0	0	*U* = 269, *z* = −1.33	0.18

^1^ Number of days on which an outcome related to smart device use was experienced in the latest week before survey. ^2^ Mann-Whitney U test, 2-tailed.
